# Global Proteomics Indicates Subcellular-Specific Anti-Ferroptotic Responses to Ionizing Radiation

**DOI:** 10.1016/j.mcpro.2024.100888

**Published:** 2024-11-29

**Authors:** Josie A. Christopher, Lisa M. Breckels, Oliver M. Crook, Mercedes Vazquez-Chantada, Derek Barratt, Kathryn S. Lilley

**Affiliations:** 1 Cambridge Centre for Proteomics, Cambridge Systems Biology Centre and Department of Biochemistry, University of Cambridge, Cambridge, UK; 2 Department of Statistics, University of Oxford, Oxford, UK; 3 Discovery Sciences, R&D, AstraZeneca, Cambridge, UK

**Keywords:** subcellular proteomics, quantitative proteomics, ferroptosis, data-dependent acquisition, Bayesian modeling

## Abstract

Cells have many protective mechanisms against background levels of ionizing radiation orchestrated by molecular changes in expression, post-translational modifications, and subcellular localization. Radiotherapeutic treatment in oncology attempts to overwhelm such mechanisms, but radioresistance is an ongoing challenge. Here, global subcellular proteomics combined with Bayesian modeling identified 544 differentially localized proteins in A549 cells upon 6 Gy X-ray exposure, revealing subcellular-specific changes of proteins involved in ferroptosis, an iron-dependent cell death, suggestive of potential radioresistance mechanisms. These observations were independent of expression changes, emphasizing the utility of global subcellular proteomics and the promising prospect of ferroptosis-inducing therapies for combating radioresistance.

## Highlights

• Protein trafficking is essential for quick cellular response to ionizing radiation.
• Subcellular proteomics detected hundreds of these potential trafficking events.
• Of these events, most were independent of total whole-cell protein expression.
• Several proteins that changed subcellular location were involved in ferroptosis.
• These changes were indicative of potential radioresistance mechanisms.

## Graphical Abstract

**Figure:**
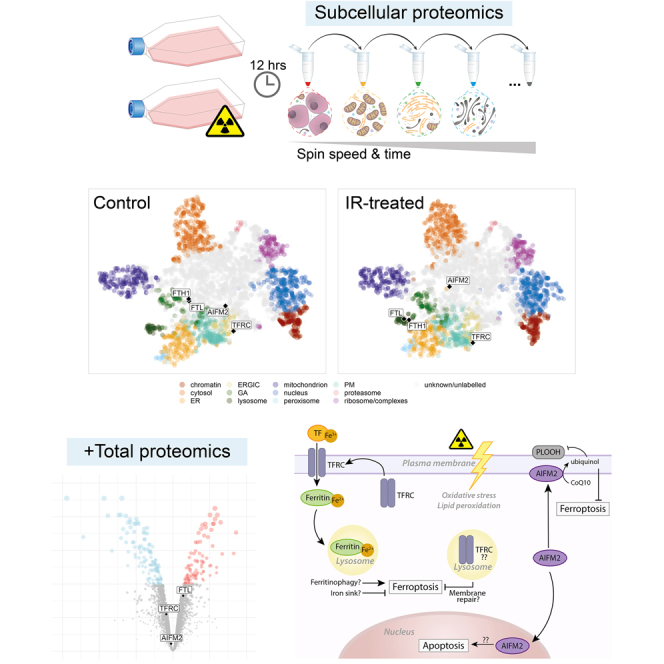

## In Brief

To explore spatial protein dynamics upon ionizing radiation, abundances of proteins in the whole-cell lysate and within subcellular compartments were measured using total and subcellular proteomics. This study was performed on a lung carcinoma model after exposure to X-rays and found hundreds of potential differential localization events independent of total expression changes. Some of these proteins were found to be key players of ferroptosis, an alternative cell death pathway, which could aid in understanding radioresistance in cancer cells.

Radiotherapy, or ionizing radiation (IR), has been a long-standing curative and palliative treatment option for cancers as well as other diseases. The high energy of IR causes a cascade of lethal biochemical reactions within cells, initiated by the ionization of water and production of free radicals, causing double-stranded breaks (DSBs) in DNA, lipid peroxidation, and resulting tissue damage (1). Whilst IR toxicity to healthy tissue is a concern, higher radiosensitivity of actively dividing and undifferentiated cells *versus* mature and non-dividing cells allows a sufficient therapeutic window for IR to be an effective cancer treatment (1). More precise delivery methods for IR, such as stereotactic ablative radiotherapy (2), have increased the therapeutic index between tumor and normal tissue response, allowing for increased doses with reduced side effects. These modern approaches have prevented IR from becoming obsolete and being superseded by molecular target–based pharmaceuticals, with approximately 50% of cancers still using radiotherapy as a component of their treatment regime (3, 4). However, naturally occurring background radiation in the environment is a chronic risk to cellular DNA. Thus, cells have evolved accordingly to have multiple DNA damage repair and antioxidant pathways to subdue the cascading effect of IR-induced free radicals (5). Therefore, as with many other oncotherapeutics, resistance to radiotherapy can be a significant issue (4).

In an attempt to improve efficacy of treatment, radiotherapy is commonly combined with therapeutics that target auxiliary pathways. For example, as the predominant mechanism of action of IR is DNA damage, there is interest in combining radiotherapy with inhibitors targeting DNA damage response pathways, such as DNA repair and cell cycle checkpoints (6). Targeting of the DNA repair pathways in cancer alone is also of high interest, as cancers are characteristically genetically unstable and generally have impaired DNA repair (*e.g.*, BRCA and ATM mutants) and thus rely more heavily on alternative repair pathways to survive during chemotherapy (7). Therefore, studying response mechanisms to IR is well-placed to elucidate the mechanisms by which tumor cells avoid cell death from DNA-damaging agents.

Proteomics approaches have rapidly accelerated our understanding of cellular biology and aided drug discovery with untargeted and high-throughput screening (8). However, generally, these approaches focus on changes in protein expression and lack spatial information—that is, where a molecule resides within a cell, its surrounding microenvironment, and its proximity to molecular interactors. This potentially overlooks key mechanisms in cellular functions, responses, and pathological mechanisms. For example, many diseases have been associated with defective protein trafficking, such as cystic fibrosis, amyotrophic lateral sclerosis, and pulmonary atrial hypertension, which may have unremarkable expression profiles of the affected proteins (9, 10, 11, 12, 13). Whilst microscopy provides such information, this technology is generally reserved for targeted experiments and suffers from limitations with gene fusion artifacts or non-specific antibodies (14, 15). Proximity labeling approaches, where a protein of interest is genetically fused with a promiscuous enzyme capable of generating activated biotin that labels its immediate subcellular environment, have also been used to map subcellular distributions of proteins (16). This approach is difficult to scale to a cell-wide method, requires genetic modification, and suffers from differential labeling in different subcellular niches leading to a biased map of subcellular residency of proteins (10, 17, 10, 17). This study used an untargeted subcellular proteomics method, Localization of Organelle Proteins by Isobaric Tagging after Differential ultraCentrifugation (LOPIT-DC) (18, 19, 20), to capture subcellular information of thousands of proteins in multiple subcellular compartments in the A549 lung carcinoma cell line after exposure to 6 Gy IR to detect trafficking-specific responses. Combining this untargeted and unbiased cell-wide approach with targeted validation, several subcellular-specific changes of proteins associated with the iron-dependent regulated cell death pathway, ferroptosis, were detected. This indicated a radio-resistant response to IR within these cells, alongside expression changes of related pathways.

## Experimental Procedures

### Experimental Design and Statistical Rationale

A multi-method approach was employed to investigate the subcellular landscape of A549 cells in response to X-ray exposure. For all experiments, three biological replicates were processed with either sham irradiation (n = 3) or irradiation (n = 3) at a dose of 6 Gy X-ray before proceeding with either flow cytometry, total proteomics, phosphoproteomics, subcellular proteomics, or imaging. Statistical tests that correspond with each experiment are detailed in the Supplemental Methods.

### Cell Culture and X-Ray Treatment

Lung epithelial carcinoma cell line, A549 (CCL-185; American Type Culture Collection), was certified as mycoplasma free. Cells were kept in culture for no longer than a month and were grown in Ham's F12 (Sigma–Aldrich), with 10% fetal bovine serum (ThermoFisher; Lot: 42F3393K) and 1% L-glutamine. Cells were passaged at around 70% confluence by washing with phosphate-buffered saline before dissociating from the flask with TrypLE Express (Gibco; 12604013) and kept at 37 °C and 5% CO_2_. A total absorbed dose of 6 Gy was administered using either a Faxitron CellRad or Pantak HF320 kV X-ray system. The Pantak HF320 kV X-ray system operated at 220 kV, 14 mA with a 0.5 Cu filter with a dose rate of 428 cGy/min. The Faxitron CellRad used a 50 Cu with a kV/mA dependent on the shelf placement in the instrument. Control cells were sham irradiated (kept in the same conditions as the IR-treated cells).

### Localization of Organelle Proteins by Isobaric Tagging After Differential ultraCentrifugation

**Fig. 1:**
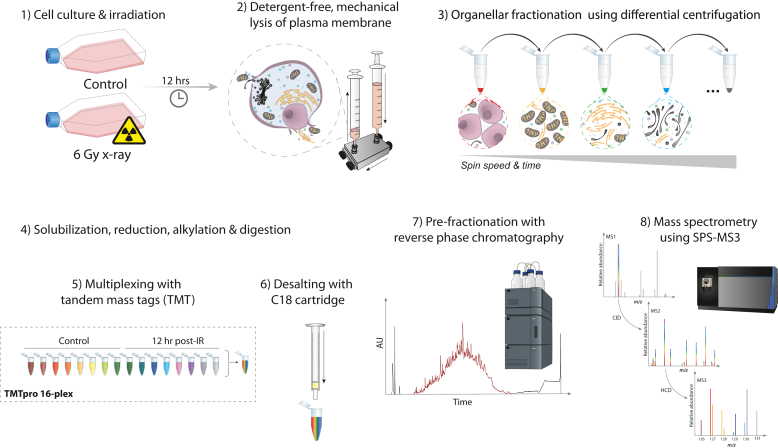
**LOPIT-DC sample preparation and LC–MS/MS workflow.** Briefly, A549 cells were either dosed with 6 Gy X-rays or sham-irradiated (control) (1), then 12 h later plasma membrane (PM) lysed using a ball-bearing homogenizer (2). Organellar-enriched pellets were achieved using differential centrifugation using a gradient of different spin speeds and time (3) before pellet solubilization using urea-based buffer and sonication followed by dithiothreitol reduction, iodoacetamide alkylation, and trypsin digestion (4). Solubilized pellets were labeled with tandem mass tags (TMTs) (5), pooled, and salts removed using C18 cartridges (6). Offline fractionation of the pooled TMT-labeled organellar-enriched sample was performed using reverse-phase chromatography (7) before analyzing using an Orbitrap mass spectrometer in SPS-MS3 mode (8). LOPIT-DC, Localization of Organellar Proteins by Isobaric Tagging after Differential ultraCentrifugation.

Performed as previously published (19). Briefly, cells were harvested, washed, and resuspended in an isotonic lysis buffer 12 h after (sham) irradiation. Cell plasma membrane (PM) lysis was performed using a ball-bearing homogenizer (Isobiotec) with a 12 μM clearance. Differential centrifugation was performed using a Beckman Optima MAX-XP ultracentrifuge (with a TLA-55, fixed angle rotor) with spin speeds and times in supplemental Table S1. Samples were always kept on ice or at 4 °C throughout the procedure. The resulting supernatant was precipitated using chilled acetone. Organellar-enriched pellets were solubilized using a urea-based buffer and sonication. Organellar-enriched lysates then underwent reduction, alkylation, digestion, tandem mass tag (TMT) labeling, clean-up, and LC–MS/MS (Fig. 1, detailed in Supplemental Methods).

### Total Proteomics Harvesting, Protein Digestion, TMT Labeling, and Clean-Up

Cells were harvested and washed 12 h after (sham) irradiation, as LOPIT-DC experimental procedure, and lysed in a urea-based buffer using sonication. LOPIT-DC and total proteomics samples were processed with the same protocols from this point on. Protein concentrations of LOPIT-DC pellets and total proteomics samples were measured using bicinchoninic acid protein assay and were normalized to 50 μg/100 μl lysis buffer. Samples underwent dithiothreitol reduction, iodoacetamide alkylation, and acetone precipitation, followed by trypsin digestion at 37 °C. TMT labeling was performed as per the manufacturer’s instructions. TMT 10-plex (ThermoFisher; 90406) were used for total proteomics sample labeling, and TMTpro 16-plex (ThermoFisher; A44520) were used for LOPIT-DC pellet labeling. Samples were lyophilized before performing solid phase extraction using SepPak C18 columns (Waters; WAT054955). Peptides were washed with 0.1% TFA, eluted with 70% acetonitrile/0.05% acetic acid, and lyophilized (detailed in Supplemental Methods).

### LC–MS/MS

Total proteomics and LOPIT-DC samples were pre-fractionated offline using reverse-phase ultra-performance liquid chromatography (UPLC) with an Acquity UPLC System with diode array detector (Waters) and an Acquity UPLC BEH 283 C18 column (2.1-mm ID × 150-mm; 1.7-μm particle size) (Water; 186002353). Peptide fractions were collected every minute of the gradient over a 50 min linear gradient resulting in 15 concatenated fractions. Samples were lyophilized and resuspended in 0.1% formic acid. Mass spectrometry (MS) analysis was performed as previously published (21) and detailed in Supplemental Methods.

### Proteomics Data Processing and Analysis

Raw MS files were processed using Proteome Discoverer 2.4 (ThermoFisher) and a Mascot server (Matrix Science), version 2.6 (22). SwissProt *Homo sapiens* database (no isoforms, 20,245 sequences, downloaded 09/04/2018) and the common repository of adventitious proteins (cRAP, version 1.0, 123 sequences) were used. An MS1 and MS2 ion tolerance of ±15 ppm and ±0.6 Da for protein identifications was used, respectively. Allowance of two missed tryptic cleavages and a minimum of two unique peptides per protein identification were set. Fixed modifications were set to carbamidomethyl (C) and TMT 10-plex or TMTpro (K, peptide N termini), and variable modifications were set to carbamyl (N-term), carbamyl (K), carbamyl (R), and deamidation (N,Q). Percolator was used to assess the false discovery rate (FDR), and only peptides with FDR <0.01 were retained (Further detailed in Supplemental Methods section).

Proteomics data were analyzed in R (v4.1.3) using the limma (v3.50.3) Bioconductor package (23). An empirical Bayes moderated *t-*test was performed for differential expression analysis of the total proteomics data (24). *p*-values were adjusted for multiple testing using the Benjamini–Hochberg method (25). Gene Ontology enrichment analysis was performed using the R package, clusterProfiler (v4.2.2) (26, 27). LOPIT-DC data was analysed using the spatial proteomics package, pRoloc (v1.34.0) (28). A semi-supervised machine learning approach, Bayesian ANalysis of Differential Localization Experiments (BANDLE), was used to classify proteins to steady-state subcellular locations, identify differential localization, and estimate the uncertainty of those predictions (29). Subcellular protein markers for this algorithm were established using existing curated markers (19, 21, 30), the bioinformatics tool COMPARTMENTS (31), and the unsupervised clustering algorithm, HDBscan (32), plus other filtering criteria documented in the Supplemental Methods. BANDLE analysis was performed as documented in the Bioconductor vignettes (https://bioconductor.org/packages/release/bioc/html/bandle.html) (detailed in Supplemental Methods section).

### Flow Cytometry

Manufacturer protocols for propidium iodide (Abcam; ab139418) and CellEvent Caspase-3/7 (Invitrogen; C10427) staining were followed for the cell cycle and cell death assays, respectively. Cells were treated with 1 μg/ml nocodazole for 18 h (as previously published (33); or 100 μg/ml sodium arsenate for 2 h as positive controls for cell arrest and cell death, respectively. Sodium arsenate was also added to compensation samples. Cells were analyzed on an Attune NxT Acoustic Focusing Cytometer.

Flow cytometry analysis was performed using the FCS Express software (De Novo Software, version 7.12). Gates were set to exclude debris and doublets from the analysis. Cell cycle analysis was performed using the MultiCycle algorithm feature in the FCS Express software, using the Dean & Jett mathematical model (34). Compensation matrixes were set using the compensation control samples. Statistical analysis and visualization for both the cell cycle and cell death assay results were performed in R using a pair-wise *t-*test and Bonferroni adjustment (detailed in Supplemental Methods section).

### Immunofluorescence Microscopy

Cells were seeded into a glass-bottomed 8-high-well μ-Slide (Ibidi; 80806), exposed to 6 Gy X-ray, and incubated for 12 h. Cells were stained with MemBrite Fix 640/60 cell surface stain (Biotium; 30097) prior to fixation and according to the manufacturer’s guidelines. Cells were then fixed with 4% paraformaldehyde and permeabilized using 0.1% Triton X-100. Primary antibodies were added at the manufacturer or optimized concentration and incubated overnight at 4 °C. Cells were washed and then incubated with corresponding IgG (H + L) conjugated with Alexa Fluor 568 (Invitrogen; A11004) or Alexa Fluor 488 (Invitrogen; A-11008) with 1:2000 dilution before adding ProLong Gold Antifade Mountant with 4′,6-diamidino-2-phenylindole (DAPI) (Invitrogen; P36931). Single-stained and secondary-only controls were prepared to assess non-specific binding or autofluorescence. See supplemental Table S2 for antibody details.

A Zeiss LSM 880 confocal microscope with a 40×/1.30 Plan Apo Oil objective lens and a Piezo stage was used for image acquisition. Fluorophores were excited using 405 nm (blue diode), 488 nm (argon), 561 nm (He 543), and 633 nm (He 633) lasers with 0.05%, 1.5%, and 3.0% power, respectively. Signal was detected using two photomultipliers and a gallium arsenide phosphide array detector in combination with filters set to 400 to 440 nm, 507 to 552 nm, 585 to 620 nm, 650 to 690 nm, to detect DAPI, AlexaFluor488, AlexaFluor568, and MemBrite Fix 640/60, respectively (detailed in Supplemental Methods section). Colocalization analyses, segmentation, and foci quantification were performed using the open-source CellProfiler software (v4.2.1; Broad Institute) (35) (detailed in Supplemental Methods section).

## Results

### DSBs and Total Proteome Level Changes Were Detected Without Marked Changes in Cell Cycle or Cell Death 12 h Post-IR

**Fig. 2:**
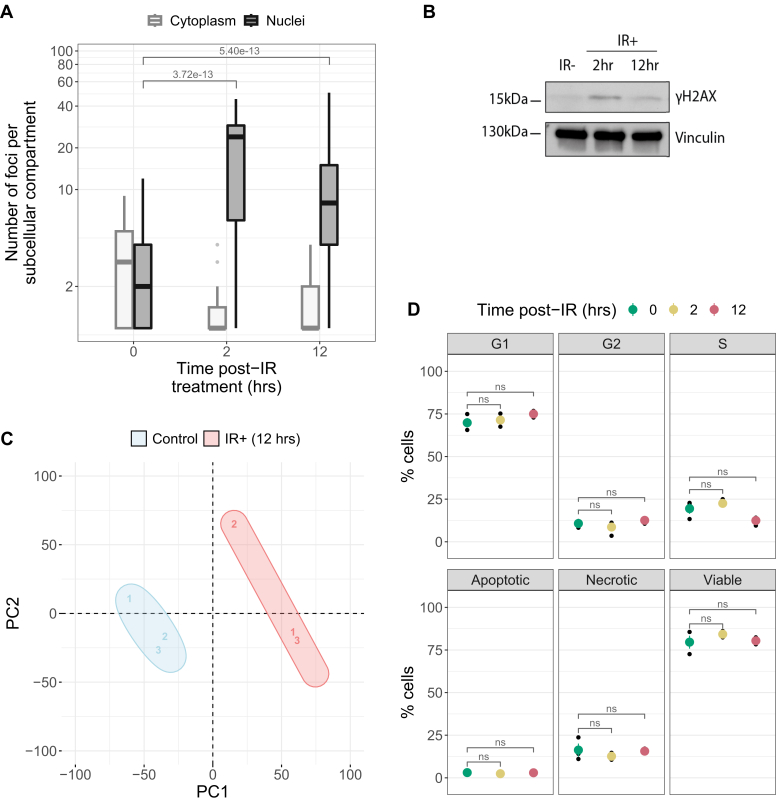
**DNA damage, cell cycle status, apoptotic status, and proteome-level changes 2 and 12 h after IR exposure*versus* sham irradiated controls.***A*, number of yH2AX foci measured in the nuclei and cytoplasm 0, 2 and 12 h post-IR exposure using quantitative confocal microscopy (n = 3), (*B*) with corresponding Western blot probing yH2AX at these time points. *C*, principal component analysis (PCA) of total proteomics data for control (*blue*) *versus* 12 h post-IR (*red*, n = 3). *D*, flow cytometry propidium iodide and CellEvent Caspase3/7 assays measuring cell cycle (*top*) and apoptotic status (*bottom*), respectively, at 0, 2, and 12 h post-IR (n = 3). IR, ionizing radiation.

At 12 h post-IR, DSBs were confirmed by immunoprobing for phosphorylated serine 139 on histone H2AX (γH2AX) and quantitative imaging for γH2AX foci in the nuclei (Fig. 2, *A* and *B*, supplemental Fig. S1 and supplemental Table S3). Cytosolic γH2AX foci measurements were also taken as a control. A large increase in nuclear γH2AX foci 12 h after X-ray exposure (*p* = 5.40e-13, r = 0.58 [0.46–0.68]) demonstrated that these cells responded typically to X-ray exposure (36). In addition, total proteomics further confirmed biological response to X-ray exposure with dominant variance in principal component analysis driven by control *versus* IR-exposed experimental groups (Fig. 2*C*). Flow cytometric measurements did not detect significant changes in cell cycle and cell death populations 12 h post-IR (Fig. 2*D* and supplemental Table S4).

### Upregulated Proteins Identify Characteristic Cellular Responses to IR

**Fig. 3:**
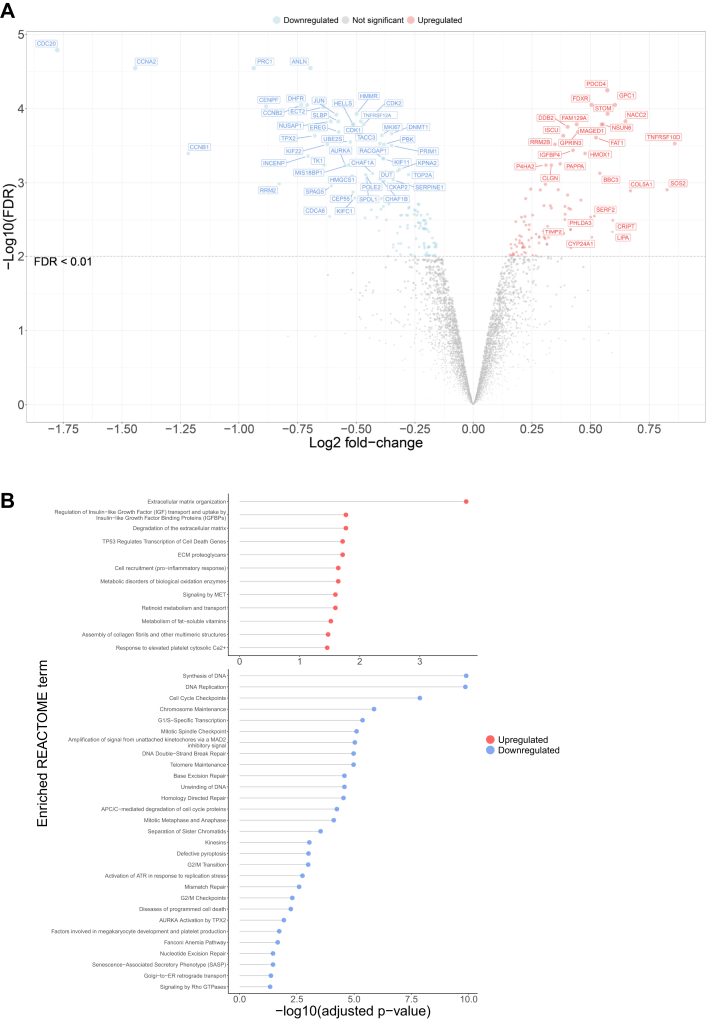
**Total proteomics data for control *versus* IR-treated A549 cells 12 h post-IR.***A*, Volcano plot of differential expression analysis for the total proteomics data in control *versus* 12 h post-IR in A549 cells. Colored points denote all proteins with FDR <0.01 and all labeled proteins with FDR <0.001 or fold change < −0.5 or >0.5. *B*, enriched REACTOME terms for downregulated and upregulated proteins (FDR <0.01) 12 h post-IR (n = 3). FDR, false discovery rate; IR, ionizing radiation.

A total of 6163 unique proteins were quantified in the total proteomics dataset, with 101 and 142 proteins upregulated and downregulated post-IR (FDR <0.01), respectively (Fig. 3*A* and supplemental Data 1). As an additional quality control, we plotted the mean protein expression of the control A549 cell samples to the A549 RNA expression data from the Broad Institute’s public DepMap database (https://depmap.org/portal, (37) and found these different -omics expression datasets positively correlated (*R* = 0.53, *p* < 2.2e16, supplemental Fig. S2). Surprisingly, many REACTOME (38) and cellular compartment terms that are enriched in the downregulated proteins are related to DNA repair, which may have expected to be upregulated after IR (Fig. 3*B*). However, many of the proteins that are involved in DNA damage repair are also involved in cell cycle progression, which is reduced or halted after IR exposure. While this observation contrasts with what was seen in the aforementioned flow cytometry analysis, this suggests proteomic-level detection of initiation of cell cycle arrest mechanisms may precede the morphological and physiological changes measured in flow cytometry (*i.e.*, differences in DNA content in cell cycle stages) (Fig. 2*D*). Phosphoproteomic analysis on these samples showed increased phosphorylation of key DNA damage repair proteins at this time point post-IR (supplemental Fig. S3, supplemental Data 2 and supplemental Methods), demonstrating the nuances of molecular signaling of DNA repair in early cellular response to IR-induced DNA damage.

Upregulated proteins were enriched for REACTOME terms, such as extracellular matrix (ECM) and retinoid metabolism. Previous studies have demonstrated that retinoids have a radioprotective effect, preventing radiation-induced apoptosis in cells (39, 40) and have been found to upregulate tissue inhibitors of matrix metalloproteinases (TIMPs) (40). Upregulation of TIMP2, alongside other proteins involved in ECM organization, such as collagens, laminins, and integrins, was detected in the total proteomics analysis (Fig. 3, *A* and *B*). Prominent upregulation of these proteins is unsurprising, as IR-related dermatitis can be induced at levels of 6 Gy or more (41). These changes in the ECM are also responsible for much of the downstream signaling and biological responses to this IR-induced damage, such as hepatocyte growth factor receptor (MET) signaling, which was also an enriched pathway (Fig. 3*B*). MET is essential for wound healing and activation of mitogen-activated protein kinase and PI3K-Akt signal transduction (42), which participate in DNA damage, oxidative stress responses, and cell fate (43, 44). In addition, upregulated proteins involved in insulin growth factor (IGF) signaling were also detected, and blockade of IGF signaling has shown to radiosensitize cells and be linked to radiation-mediated regulation of Ku86 *via* p38 (45, 46). Interestingly, an enriched cellular component was membrane (or lipid rafts), sphingolipid-rich membrane domains that compartmentalize signaling processes within the cell (supplemental Fig. S4). Prosurvival signaling pathways such as PI3K-Akt and IGF signaling, which cancer cells are known to hijack, can be modulated *via* lipid rafts (47).

### LOPIT-DC Captures the Dynamics of the Subcellular Proteome Post-IR

**Fig. 4:**
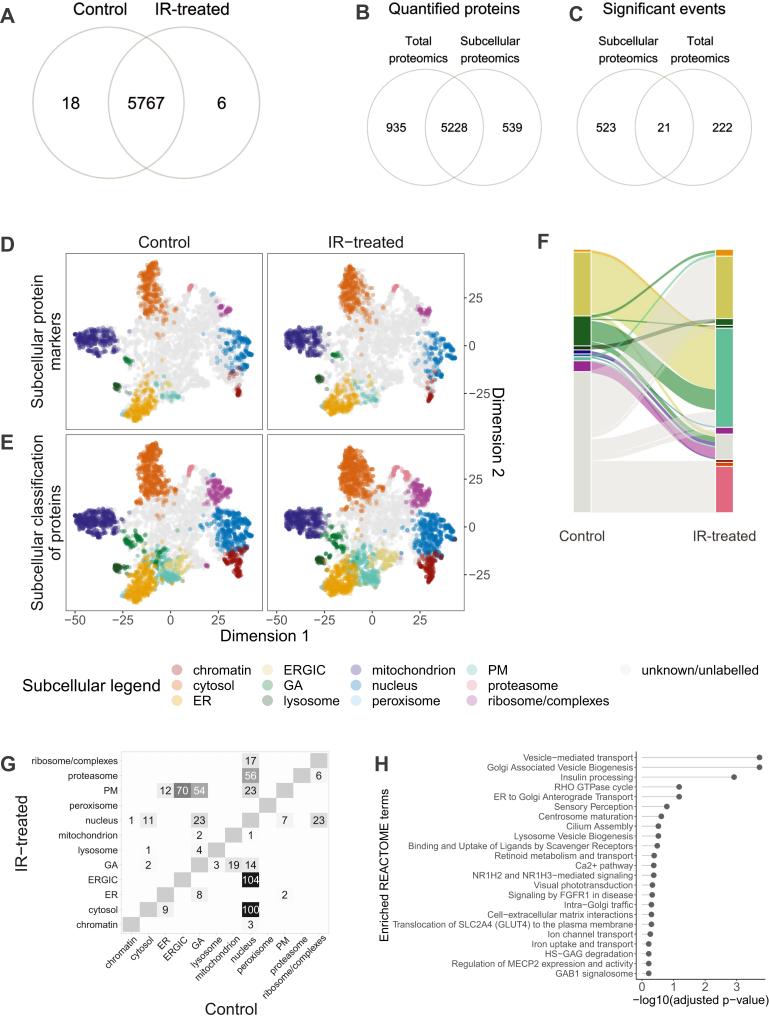
**Subcellular proteomics using LOPIT-DC to identify changes in cellular location of proteins 12 h after IR exposure.***A*, Venn diagrams of shared protein identifications between the control and IR-treated groups in LOPIT-DC experiment. *B*, the quantified protein identifications in the total proteomics data *versus* subcellular proteomics data, and (*C*) proteins that were considered significant in the LOPIT-DC data (eFDR <0.05, n = 3) and the total proteomics data (FDR <0.01, n = 3). *D*, combined t-distributed Stochastic Neighbor Embedding (t-SNE) projections of the LOPIT-DC control and IR-treated replicates, where each point represents an individual protein identification. Each color denotes the protein markers of 12 distinct subcellular compartments and (*E*) the subcellular classification of proteins using these protein markers with BANDLE. *F*, Sanky plot representing the 544 proteins identified as differentially localized after IR exposure by BANDLE, and (*G*) corresponding heatmap with numbers representing number of proteins localizing between these subcellular compartments between control and IR-treated groups. Heatmap shows the subcellular classification irrespective of whether the classification was considered an outlier/“unknown” classification. *H*, enriched REACTOME terms for proteins identified as differentially localizing. BANDLE, Bayesian ANalysis of Differential Localization Experiments; eFDR, estimated FDR; ER, endoplasmic reticulum; ERGIC, endoplasmic reticulum–Golgi intermediate compartment; FDR, false discovery rate; GA, Golgi apparatus; IR, ionizing radiation; LOPIT-DC, Localization of Organelle Proteins by Isobaric Tagging after Differential ultraCentrifugation; PM, plasma membrane.

LOPIT-DC datasets were collected in biological triplicate, and the resulting data were processed as in (19) (supplemental Data 3). Approximately 85% of proteins quantified in each LOPIT-DC sample were detected across all replicates (n = 3), with 5785 and 5773 proteins found in control and IR-treated samples, respectively, and 5767 proteins shared across all LOPIT-DC samples (Figs. 4*A* and supplemental Fig. S5). The consistency of the protein identifications, correlation profiles, and principal component analysis demonstrated the high reproducibility of these experiments (supplemental Figs. S6 and S7, *A* and *B*). A semi-supervised machine learning approach, BANDLE (29), with 1509 manually and computationally curated subcellular protein markers from previous studies, databases, and literature (Fig. 4*D*), classified 1913 and 2179 proteins to 12 subcellular classes within the control and IR-treated groups, respectively (Fig. 4*E*). BANDLE was also used to calculate which proteins had differentially localized post-IR exposure. The term “differentially localized” is to describe proteins that appear to have changed localization after stimuli based on LOPIT-DC analysis, though this does not rule out that a protein has changed in abundance in one compartment over another. Therefore, the term “trafficking” or “translocation” is avoided.

BANDLE also identified 544 differentially localized proteins (estimated false discovery rate [eFDR] <0.05) (Fig. 4*F*). The majority of the proteins that were identified as differentially localizing are transported between organelles linked to the secretory pathway and cytoskeletal remodeling, with enriched REACTOME terms such as “Golgi associated vesicle biogenesis” and “RHO GTPase,” respectively (Fig. 4, *G* and *H*). It is worth noting that the Golgi apparatus is known to fragment or disperse in response to IR, and it is even thought this fragmentation participates in DNA repair signaling *via* DNA-PKcs, increasing cell survival (48, 49). These data also suggest increased vesicle transport to the PM. For example, exocyst complex proteins, which participate in tethering secretory vesicles to the PM, differentially localized to the PM post-IR (supplemental Fig. S8) (50). Terms that are non-specific to the secretory pathway and protein trafficking were also enriched, such as “binding and uptake of ligands by scavenger receptors,” “metabolism of fat-soluble vitamins,” “iron uptake and transport,” and “transferrin endocytosis and recycling” (Fig. 4*H*). Interestingly, a lot of these terms and the proteins themselves are linked to lipid and iron metabolism, indicating subcellular-driven responses to IR-induced lipid peroxidation (51, 52).

### Little Overlap Between Differentially Localized and Differentially Expressed Proteins

Of the 5228 proteins that were quantified in both the LOPIT-DC and the total proteomics experiments, only 21 proteins were found to be differentially expressed (FDR <0.01) and localized (eFDR <0.05) after IR (Fig. 4, *B* and *C*). This suggests that 96% of the proteins that were considered to have differentially localized (eFDR <0.05) were likely protein trafficking events, rather than driven by a change in abundance and accumulation of a protein in a particular subcellular compartment. However, at this time point, this could also represent newly synthesized proteins that have trafficked to a different localization to where their older degraded counterparts were predominantly localized.

While differentially regulated proteins largely differed between the total proteomics and LOPIT-DC experiments, similar or connected REACTOME terms were enriched between these datasets (Figs. 3*B* and 4*H*). Proteins from the same or similar REACTOME pathways were detected as changing in expression or localization but infrequently both, demonstrating the complementary nature of using these two different untargeted proteomics methods (supplemental Fig. S9). Proteins upregulated in the total proteomics data had overlapping terms to the differentially localized proteins, which included lipid and retinoid metabolism, as well as pathways linked to IGF signaling and ECM organization, such as GRB2-associated binder 1 signaling and heparan sulfate glycosaminoglycans signaling, respectively (Figs. 3*B* and 4*H*) (53, 54). These biological processes and pathways are all highly interconnecting and biologically relevant to IR damage and response.

Using the Human Protein Atlas (HPA) subcellular database (https://www.proteinatlas.org/) (55), we compared the proteins that were considered to have “Enhanced” or “Supported” localization status and matched these to the proteins we identified as differentially expressed or localized within our total proteomics and LOPIT-DC experiments, respectively. A smaller proportion of proteins identified as differentially localized by the subcellular proteomics analysis are documented to have a single subcellular location by HPA *versus* the proteins identified as differentially expressed by the total proteomics analysis. Also, proteins identified as differentially localized in LOPIT-DC are more confidently documented as multi-localized proteins by HPA (supplemental Fig. S10). However, there is still a large proportion (∼34%) with a single location in the LOPIT-DC data. This may be due to the strict criteria HPA use for defining location of proteins, which may overlook some multi-localized proteins when analyzing just the categories with higher confidence data, or these proteins have yet to be documented in multiple compartments. Also, HPA microscopy data are single-cell measurements, so more subtle multi-localizations could be missed with LOPIT-DC.

### LOPIT-DC Detected Subcellular-Specific Changes to Key Ferroptosis Proteins

**Fig. 5:**
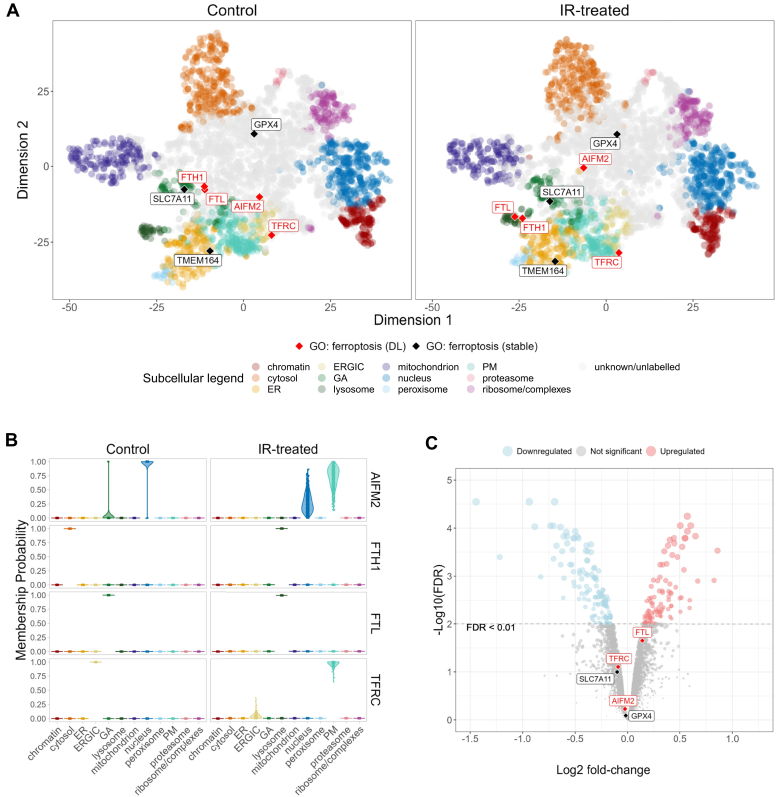
**Ferroptosis proteins in the subcellular and total proteomics data.***A*, t-SNE projections of control *versus* IR-treated labeled with proteins with associated Gene Ontology (GO) term “ferroptosis.” *Red* and *blacklabels* are proteins that were either identified as differentially localized (DL) or having a stable localization, respectively, after IR by BANDLE (eFDR <0.05). *B*, posterior predictive distributions (membership probability) across the 12 subcellular compartments for proteins identified as differentially localized and part of ferroptosis pathway in control *versus* IR-treated LOPIT-DC samples (n = 3). *C*, Volcano plot of the total proteomics data annotated with measured ferroptosis-associated proteins. BANDLE, Bayesian ANalysis of Differential Localization Experiments; eFDR, estimated FDR; ER, endoplasmic reticulum; ERGIC, endoplasmic reticulum–Golgi intermediate compartment; GA, Golgi apparatus; IR, ionizing radiation; LOPIT-DC, Localization of Organelle Proteins by Isobaric Tagging after Differential ultraCentrifugation; PM, plasma membrane; t-SNE, t-distributed stochastic neighbor embedding.

**Fig. 6:**
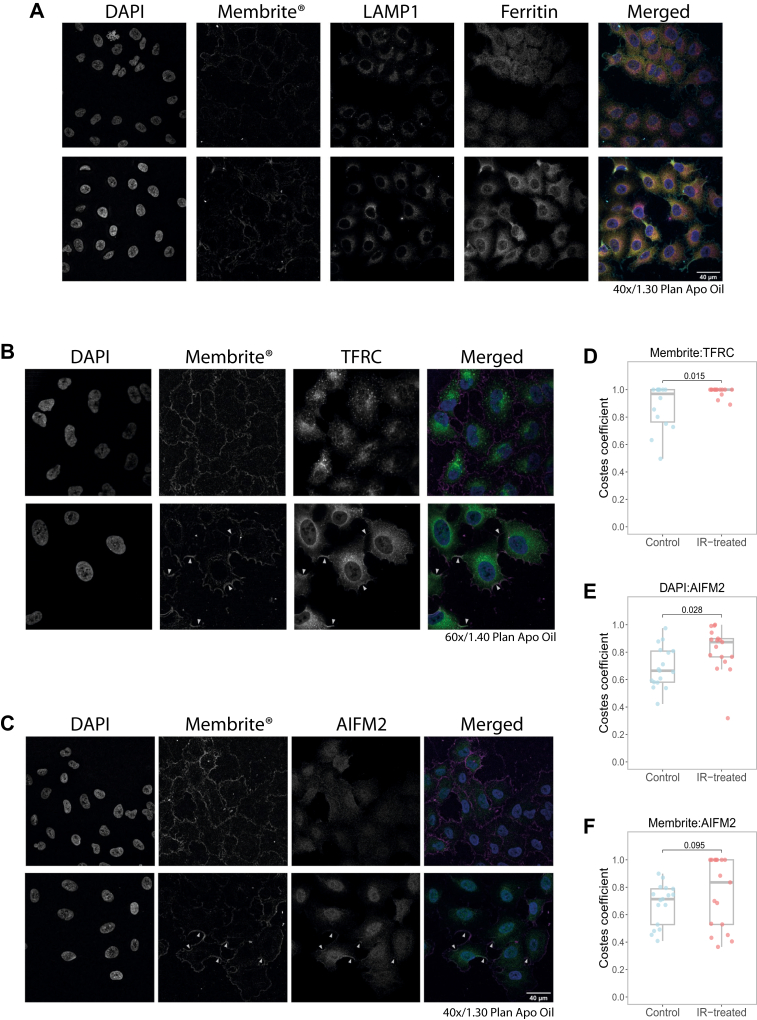
**Validation of subcellular localization of ferroptotic protein using confocal microscopy in control *versus* 12 h post-IR.** Confocal images of A549 cells control *versus* 12 h post-IR with immunostaining of (*A*) ferritin (*yellow*), with lysosomal LAMP1 (*magenta*), and PM MemBrite stain (*cyan*); (*B*) TFRC (*green*), with PM MemBrite stain (*magenta*); (*C*) AIFM2 (*green*), with PM MemBrite stain (*magenta*). All images use nucleic DAPI staining (*blue*). Corresponding quantitative microscopy analysis to assess single-cell colocalization of (*D*) MemBrite and TFRC, (*E*) DAPI and AIFM2, and (*F*) MemBrite and AIFM2 (n = 3). AIFM2, apoptosis-inducing factor homologous mitochondrion-associated inducer of death 2; DAPI, 4′,6-diamidino-2-phenylindole; IR, ionizing radiation; LAMP1, lysosome-associated membrane glycoprotein 1; PM, plasma membrane; TFRC, transferrin receptor C.

In addition to the aforementioned observations relating to lipid metabolism, retinoid metabolism, and scavenger receptors, subcellular changes to several key proteins that are part of the iron-dependent cell death pathway, ferroptosis, were observed (56, 57). These proteins included transferrin receptor C (TFRC), the heavy and light chains of ferritin (FTH1 and FTL), and apoptosis-inducing factor homologous mitochondrion-associated inducer of death 2 (AIFM2) (Fig. 5, *A* and *B*). However, in recent years, AIFM2 has been renamed ferroptosis suppressor protein (FSP1) because of its significant role in glutathione (GSH)-independent inhibition of ferroptosis (58, 59). AIFM2, TFRC, and FTL did not change in total abundance according to the total proteomics data, suggesting probable protein trafficking events (Fig. 5*C*). FTH1 was not detected in the total proteomics analysis. The subcellular localization of these proteins was verified using confocal microscopy (Fig. 6).

Within the LOPIT-DC data, AIFM2 was classified as “unknown” in both the control and IR-treated groups, suggesting a multi-localized distribution within the cell (Fig. 5*A*). However, application of the BANDLE algorithm showed a shift of steady-state localization from a nuclear/Golgi profile to a more PM/nuclear profile (Fig. 5*B*). AIFM2’s primary unperturbed steady-state localization has previously been observed in the Golgi (60) and demonstrated to exert its ferroptosis suppressor function *via* PM and lipid droplet–targeted translocation (58). Interestingly, two proteins that are part of the glutathione-dependent ferroptosis inhibition pathway, glutathione peroxidase (GPX4) and transporter solute carrier family 7 member 11 (SLC7A11) (61), appeared to remain static and lack changes in expression post-IR (Figs. 5, *A* and *C* and supplemental S11*A*). This combination of observations suggest A549 cells have a preference for glutathione-independent suppression of ferroptosis to avoid cell death upon IR exposure. Subcellular classification of AIFM2 was validated using confocal imaging and quantitative colocalization analysis comparing AIFM2 staining to nucleic DAPI and PM MemBrite staining. Control-AIFM2 was observed to be dispersed throughout the cells and then localized more discreetly to the nuclei and PM post-IR (Fig. 6*C*). Small increases in quantitative colocalization of AIFM2 with DAPI and MemBrite post-IR were measured, with median Costes coefficient (62) increased by 0.208 (*p* = 0.028, r = 0.46 [0.09–0.88]) and 0.121 (*p* = 0.095, r = 0.33 [0.02–0.68]), respectively (Fig. 6, *E* and *F*). Whilst currently in contention because of the novel role of AIFM2 in ferroptosis inhibition, nuclear AIFM2 has been previously linked to caspase-independent apoptosis (63, 64). Specifically, adduction of the lipid peroxidation product, 4-hydroxy-2-nonenal, induced AIFM2 nuclear translocation from the mitochondria to the nuclei as part of pro-apoptotic signaling (65). This evidence suggests A549 cells’ preference for caspase-independent apoptosis over a ferroptotic cell death.

**Fig. 7:**
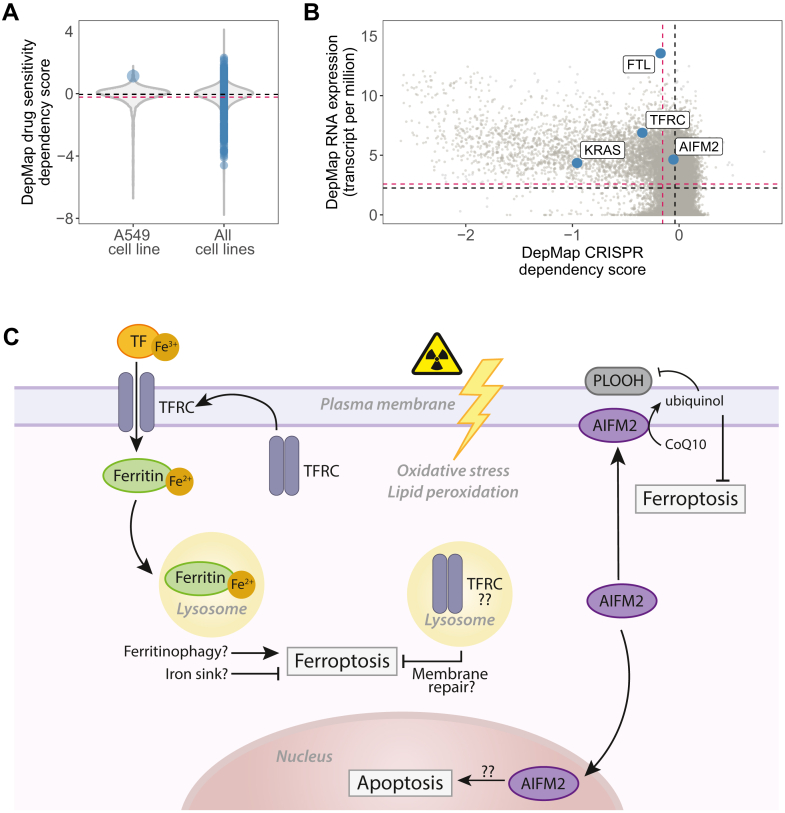
**Meta-analysis from DepMap dependency database and overview of this study’s key findings.***A*, DepMap drug sensitivity scores for all drugs in A549 cells *versus* all cell lines within the database with drug sensitivity scores for erastin plotted in *blue*. *B*, DepMap RNA expression (transcripts per million) *versus* DepMap CRISPR dependency score in the A549 cell line. *C*, schematic of key findings and potential implications within the ferroptotic pathways.

LOPIT-DC data also supported the ferritin chains, FTH1 and FTL, as being differentially localized to the lysosome post-IR (Fig. 5, *A* and *B*). Confocal microscopy of ferritin and the lysosomal marker, lysosome-associated membrane glycoprotein 1 (LAMP1), was performed to validate the subcellular proteomic findings. Visual observations showed a dispersed staining of ferritin in the control samples with increased staining around the perinuclear area post-IR, which mimics the staining pattern seen by LAMP1 in both the control and IR-treated samples (Fig. 6*A*). Despite these visual observations, the corresponding colocalization analysis was shown to not be significant (supplemental Fig. S12). Previous work using the A549 cell line has found significantly higher content of ferritin than a non-tumorigenic epithelial lung cell line counterpart and suggested intralysosomal ferritin provided a chelating function to maintain lysosomal membrane stability, preventing lysosomal leakage and subsequent autophagy, apoptosis, and necrosis (66). If autophagy is activated, ferritin is degraded by the lysosomes causing the cascade of cell death responses characteristic of ferroptosis (67, 68). This process is known as ferritinophagy, which increases the bioavailability of iron in the cell, subsequently increasing sensitivity to ferroptosis (69). Therefore, within the data presented here, it is difficult to determine whether the cells 12 h post-IR are undergoing active ferritinophagy, increasing ferroptosis sensitivity, or are chelating the endogenous iron to protect the cells from ferroptosis (Fig. 7*C*).

In addition, in our LOPIT-DC data, TFRC was found to differentially localize from endoplasmic reticulum–Golgi intermediate compartment (ERGIC) to the PM upon IR exposure (Fig. 5, *A* and *B*) (70). Validation *via* imaging confirmed this with increased staining and colocalization of MemBrite and TFRC (*p* = 0.015, r = 0.60 [0.18–0.81]) (Fig. 6, *B* and *D*). Interestingly, control-TFRC appeared to have asymmetric perinuclear staining with this staining becoming more dispersed and evenly distributed around the perinucleus post-IR, similar to LAMP1 staining (Fig. 6, *A* and *B*). This suggests ERGIC or Golgi apparatus (GA) localization within the control samples *versus* more active trafficking with potential lysosomal localization of TFRC post-IR. TFRC is a surface receptor, which internalizes transferrin–Fe^3+^ complexes *via* endocytosis and a significant regulator of intracellular iron. Increased intracellular iron is a key mediator of ferroptosis, and increased expression of TFCR *via* mitogen-activated protein kinase kinase signaling in cancer cells has been attributed to higher sensitivity of some cancer cells to ferroptosis (71, 72, 71, 72). Furthermore, PM-localized TFRC has recently been suggested as a reliable biomarker for ferroptosis, indicating that this observed increase in PM-targeting of TFRC could be sensitizing cells to ferroptosis (70). However, in our imaging data, the additional dispersed staining of TFRC visually appeared similar to LAMP1 staining post-IR and could also suggest an opposing anti-ferroptotic mechanism based on the following previous literature. In stress conditions, TFRC has been observed to shift its localization further down the endocytic pathway to the lysosomes. It is thought that TFRC is involved in recruitment of galectin-3 (LGALS3) to damaged lysosomes where it participates in endosomal sorting complexes required for transport (ESCRT)-III-mediated membrane repair and/or autophagy of damaged lysosomes. Lysosomal TFRC recruitment of LGALS3 is thought to favor ESCRT-III-mediated repair over autophagy (73). This suggests a cell survival mechanism, rather than a pro-ferroptotic mechanism (Fig. 7*C*). To assess this, a further co-staining experiment was performed using LAMP1 as a lysosomal marker, but colocalization analysis of LAMP1 and TFRC post-IR showed no significance (supplemental Fig. S13). Further investigation with more optimized conditions is required to confirm this hypothesis.

Another observation with an interconnecting function with ferroptosis was the differential localization of hereditary hemochromatosis protein (HFE), which was found to localize to the PM after IR exposure (supplemental Fig. S11*B*). HFE is known to dimerize with TFRC at the PM to regulate iron intake by reducing TFRC affinity to transferrin (74, 75). Generally, HFE has been studied in the context of hemochromatosis, where its most common mutation HFE (C282Y) prevents cell surface expression, destabilizing iron homeostasis and leading to iron overload in tissues, particularly the liver (75, 76, 77). This finding suggests the cells are attempting to stabilize cellular iron levels and, therefore, an anti-ferroptotic response.

In the DepMap database (37), the lower the dependency score the more a cell line is dependent on that gene or drug. When assessing the A549 cell line against all cell lines tested in DepMap, A549 cells had a particularly high drug sensitivity dependency score to erastin, a ferroptosis inducer (Fig. 7*A*), suggesting resistance to ferroptosis induction. Also, we annotated these data with our proteins of interest (AIFM2, TFRC, and ferritin [FTL]) in relation to Kirsten rat sarcoma viral oncogene homolog (KRAS), the mutant protein that drives oncogenesis in A549 cells. Interestingly, ferritin (FTL) has particularly high RNA expression within A549 cells, which corresponds to previous literature where high ferritin levels have been found in A549 cells (37, 66, 37, 66). While AIFM2 and FTL have a relatively central distribution around the median (*dotted black line*) and mean (*dotted red*) dependency scores, respectively, suggesting these are non-essential for A549 cell survival, TFRC appears to have a relatively low dependency score (as KRAS) (Fig. 7*B*). This suggests A549 cells are more sensitive to cell death after CRISPR knockout of TFRC. These CRISPR screens are performed on unperturbed cells; therefore, AIFM2 may not be essential in the absence of IR. However, it is interesting to see the relationship of TFRC and ferritin in this plot and could further support the theory that TFRC provides a protective effect against naturally high iron levels in the cell (Fig. 7*C*).

## Discussion

Within this study, a combination of untargeted and targeted techniques were exploited to gain an increased insight of the expression and subcellular dynamics of proteins upon IR exposure, identifying 243 and 544 differentially expressed and localized proteins, respectively. Of these, only 21 of the proteins measured were deemed to both change in abundance and subcellular localization, highlighting the complementary nature of these techniques and epitomizing the utility of using untargeted subcellular proteomics methods. Whilst proteins that changed in abundance or localization differed, the enriched pathways were highly interlinked, such as iron metabolism and ECM organization.

A subset of proteins that appeared to have subcellular-specific changes within our proteomics data and are involved in ferroptosis-related signaling—ferritin (FTH and FTL1), TFRC, and AIFM2 - were further investigated using a targeted imaging approach. Lately, ferroptosis has come under the research spotlight, because of increased interest as a potential drug target for diseases such as cancer (78, 79). This iron-dependent cell death response is characterized by induction of lipid peroxidation and iron overload. Our data suggest a prominent response to IR-induced lipid peroxidation and cellular iron homeostasis. Using these observations alongside prior literature, several hypotheses as to the function of these potential translocation events in relation to ferroptosis have been made (Fig. 7*C*).

While PM-targeted TFRC suggested a pro-ferroptotic response to IR (70), the concurrent localization of HFE to the PM indicated dimerization of these two proteins and subsequent regulation and prevention of iron overload in the cells (74, 75). In addition, visual observations of the TFRC staining pattern in confocal microscopy appeared to mimic the distribution of LAMP1 staining. Lysosomal localization of TFRC has previously been suggested to participate in ESCRT-III-mediated membrane repair and therefore another protective mechanism (73, 80). LOPIT-DC data showed both ferritin chains localized to the lysosomes post-IR. It has previously been shown that A549 cells have unusually high levels of ferritin compared with a non-tumorous counterpart, and it has been hypothesized that the lysosomes are used as an iron sink to protect cells (66). This enhanced lysosomal integrity and lysosomal iron chelation capabilities could explain the challenge in treating lung tumors (81). However, lysosomal ferritin can also induce ferritinophagy, a ferroptosis-specific autophagy (82). Finally, AIFM2, also becoming more commonly known as ferroptosis suppressor protein 1 (FSP1), was found to localize to the PM upon IR. This is a characteristic translocation indicating suppression of ferroptosis *via* the antioxidant, glutathione-independent AIFM2–CoQ_10_ axis (58, 59). Interestingly, proteins from the orthogonal glutathione-dependent cyst(e)ine–GSH–GPX4 axis remained unchanged in both abundance and subcellular localization, indicating a potential preference or reliance of these cells to the AIFM2–CoQ_10_ axis for ferroptosis suppression.

A549 cells harbor a KRAS mutation. RAS oncogenes have been implicated in radioresistance and found to be resistant to ferroptosis inducers, such as GPX4 inhibitors (83). Recent research has also shown elevated levels of AIFM2 in KRAS mutant tumors *versus* non-tumorous tissue, with elevated levels linked to tumor initiation and ferroptosis resistance (84). A549 cells are also kelch-like ECH-associated protein 1 (KEAP1) mutants, which has recently been demonstrated to play a role in ferroptosis resistance *via* lack of inhibition of the AIFM2 transcription factor, nuclear factor erythroid 2-related factor 2 (NRF2). Inhibition of AIFM2 in combination with IR markedly increased radiosensitivity within these cells compared with IR or AIFM2i alone (85).

These findings demonstrate the benefits of generating subcellular cell-wide proteomic maps for gaining insights into protein function, particularly those with multifactorial roles, which would otherwise be missed when assessing expression alone. The recent wave of new findings in the ferroptosis community, along with the findings in this study, solidifies the need for further research into the druggability of this cell death pathway in combination with current therapeutic options (79).

## Data Availability

There is a dedicated R Shiny app for interactive viewing of the subcellular proteomics data online at http://proteome.shinyapps.io/a549lopit2024. All proteomics data have been deposited to the ProteomeXchange Consortium *via* the PRIDE partner repository (86) with the dataset identifier PXD055123. Information related to the raw data files can be found in the Supplemental Data.

## Supplemental data

This article contains supplemental data (21, 24, 25, 26, 27, 28, 29, 30, 31, 32, 33, 34, 35, 37, 55, 87, 88, 21, 24, 25, 26, 27, 28, 29, 30, 31, 32, 33, 34, 35, 37, 55, 87, 88, 37, 55, 87, 88).

## Conflict of interest

The authors declare no competing interests.
