# Supplementary material for: Global Proteomics Indicates Subcellular-Specific Anti-Ferroptotic Responses to Ionizing Radiation
Source: Mol Cell Proteomics. 2024 Nov 29;24(1):100888. doi: 10.1016/j.mcpro.2024.100888 (PMC11780130; doi:10.1016/j.mcpro.2024.100888)
Supplement: Supplemental Figures S1–S13 [file mmc2.docx]

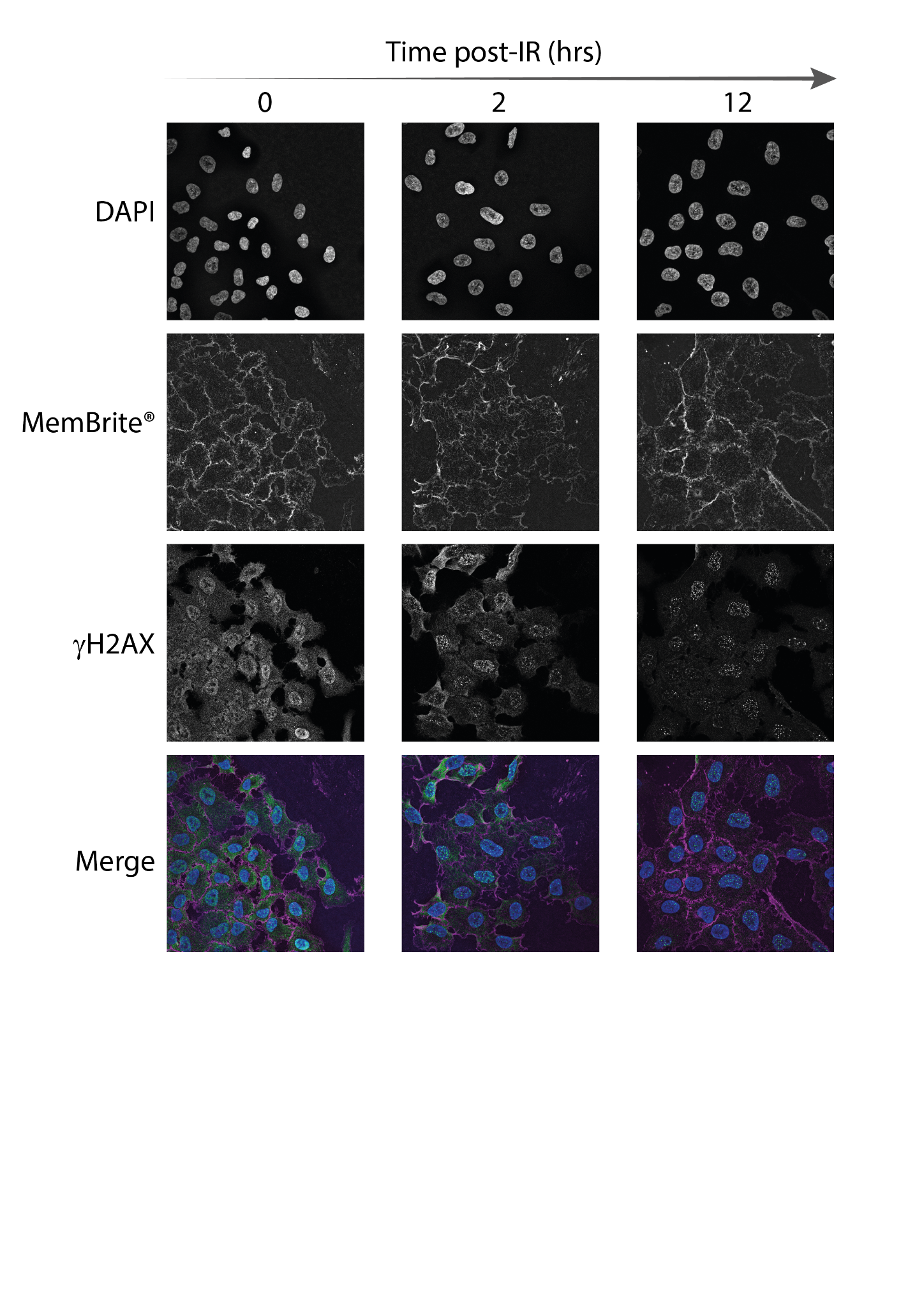


**Figure S1.** Examples of microscopy images used for measuring number of ƴH2AX nuclear and cytoplasmic foci at 0, 2 and 12 hrs post-IR. DAPI was used as a nuclear stain and MemBrite® as a PM stain to distinguish cell boundaries. Colour image shows all three channels merged into one image (DAPI: blue, MemBrite®: magenta, ƴH2AX: green).

**Figure S2.** Correlation between the mean protein expression derived from our total proteomics data within the control A549 cell samples against A549 cell RNA expression of the corresponding gene transcripts from the Broad Institute DepMap database (Tsherniak et al., 2017). Blue line denotes a linear fit (y ~ x) with *R* and *p*-value calculated using Pearson.
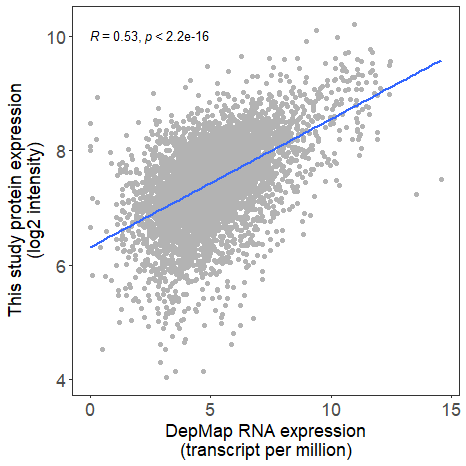


**A**

**
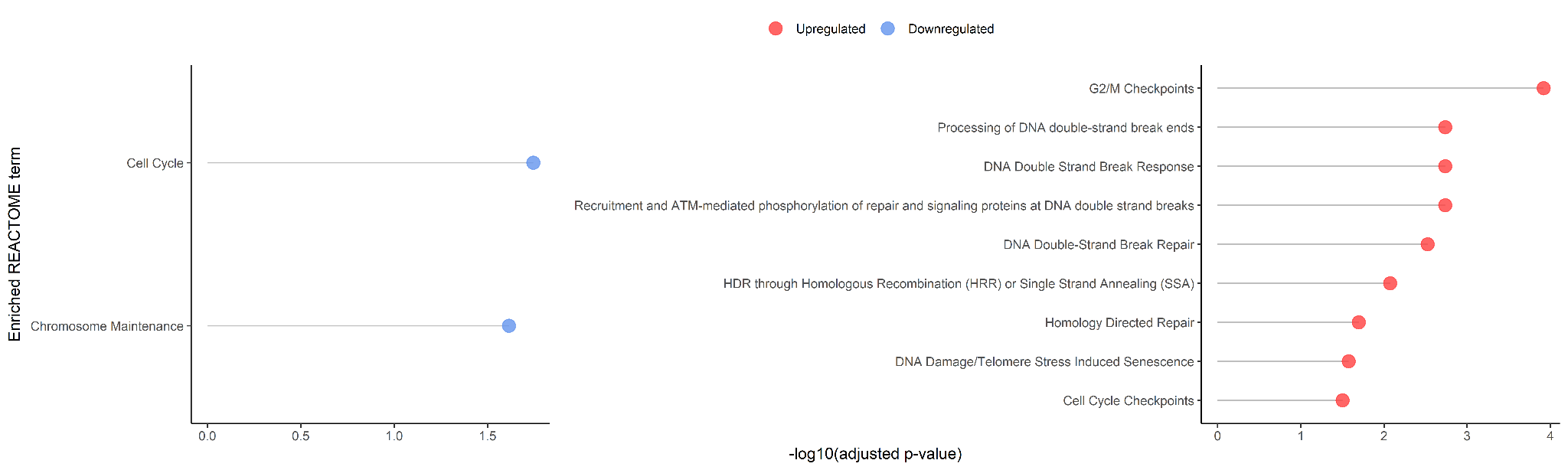
**

**B**

**
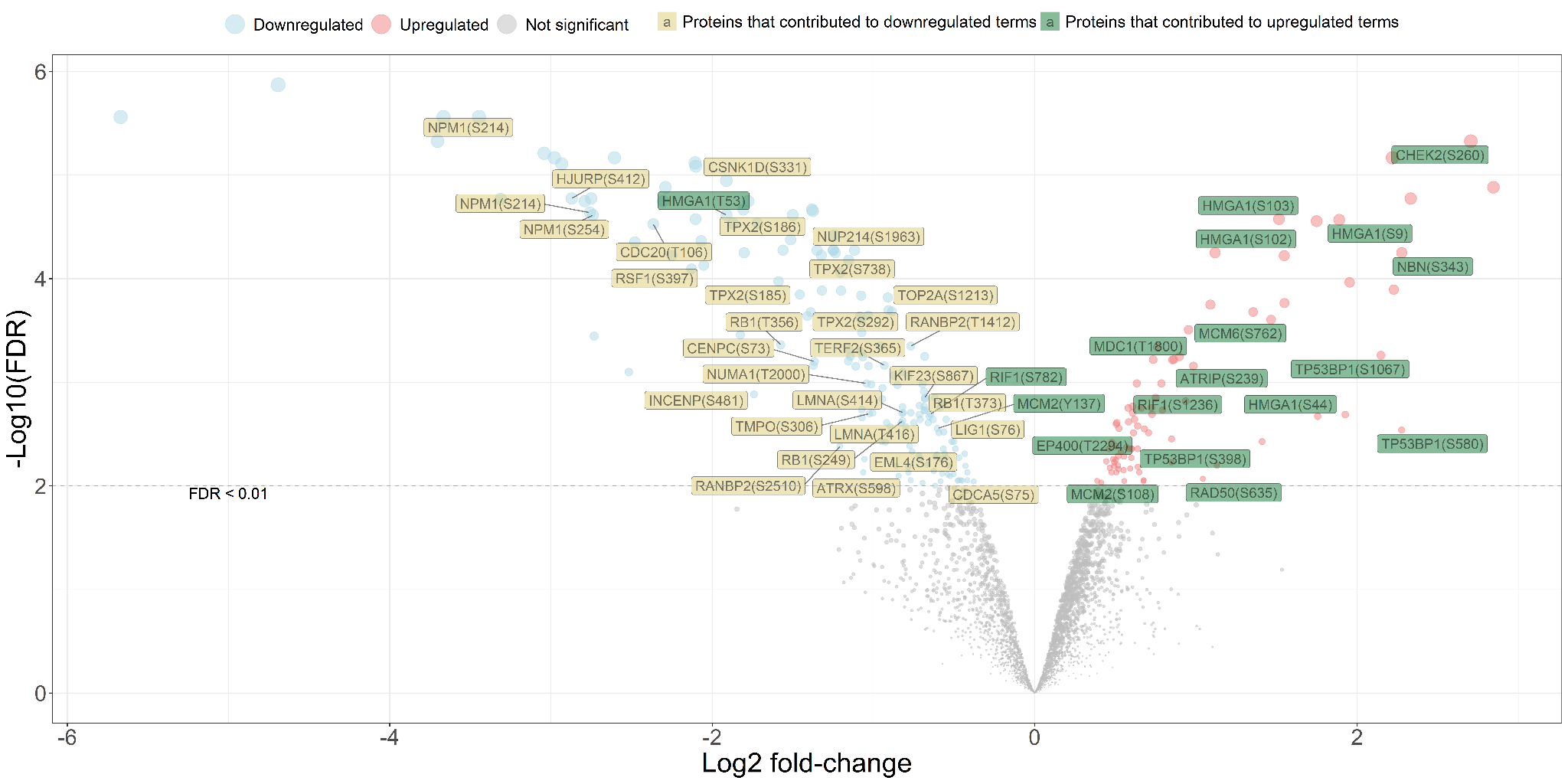
**

**Figure S3.** Corresponding phosphoproteomics data of same samples that underwent total proteomics analysis. (A) Enriched REACTOME terms for the proteins the up- and down-regulated phosphopeptides derive from at 12 hrs post-IR, and (B) these phosphosites annotated on volcano plot from differential expression analysis of phosphoproteomics data.

**Figure S4.** Enriched biological process (BP) and cellular component (CC) gene ontology terms for down- and up-regulated proteins (FDR < 0.01) 12 hrs post-IR in the total proteomics data.
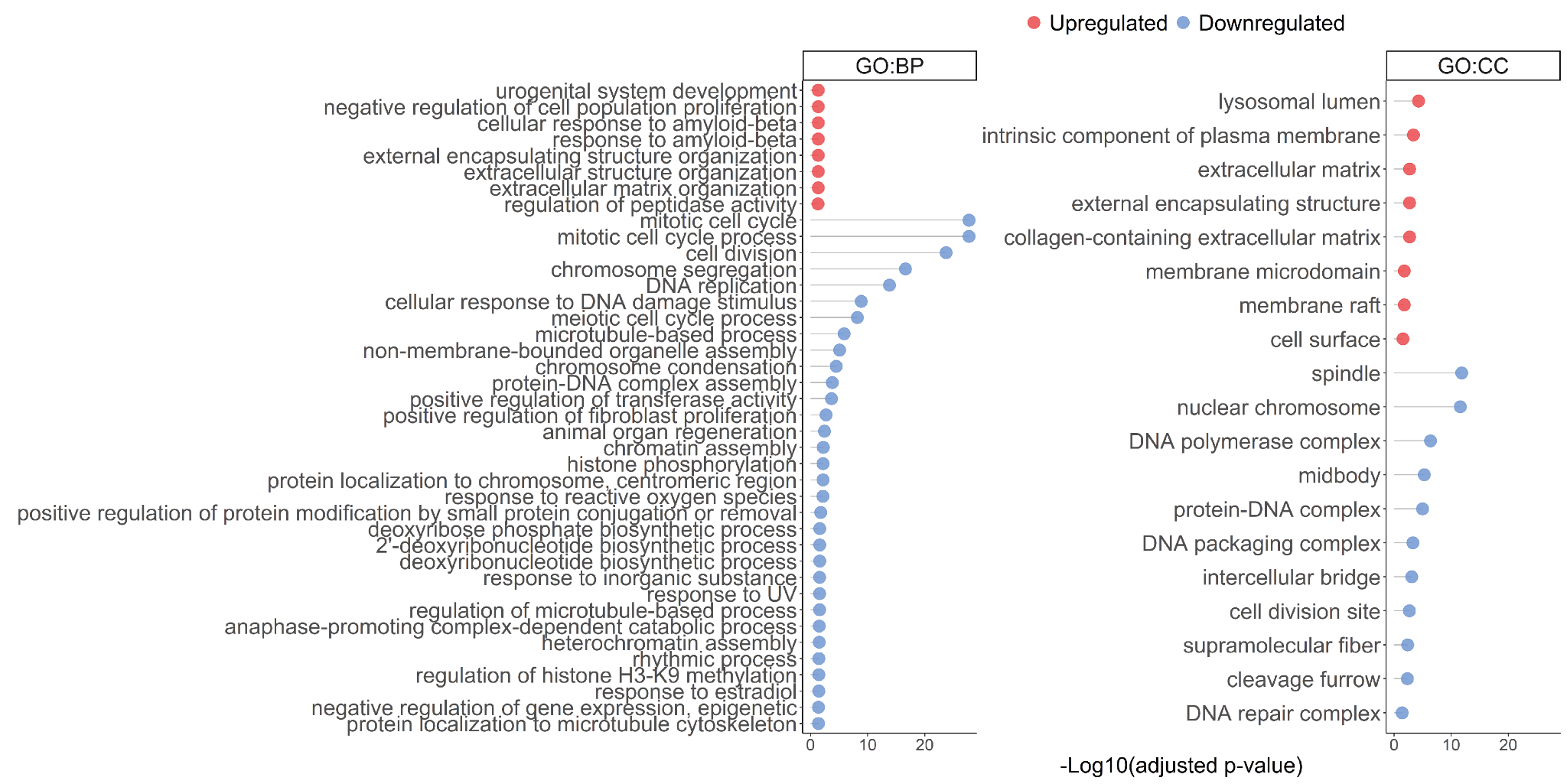


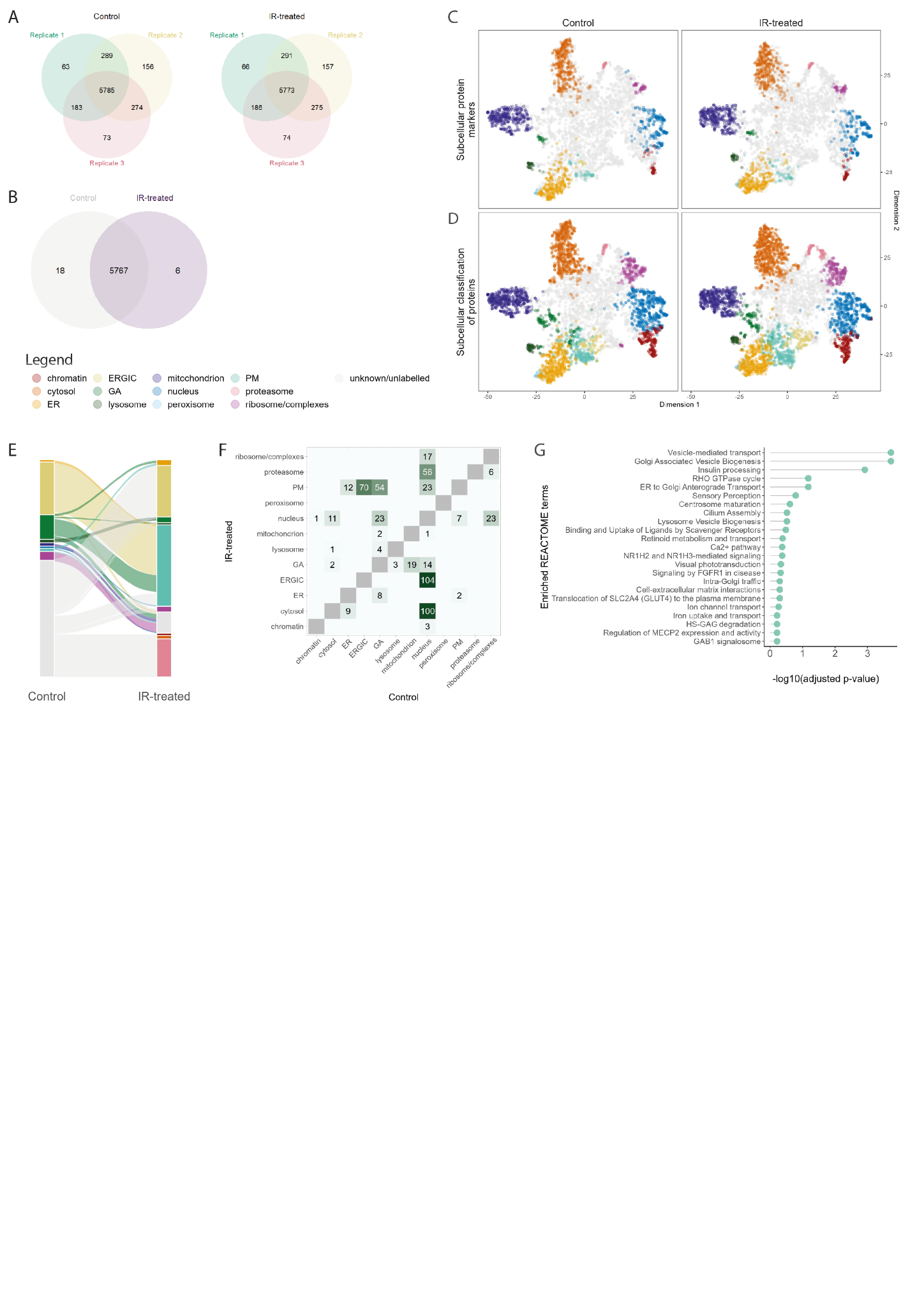


**Figure S5.** Venn diagrams demonstrating overlap of protein identifications between LOPIT-DC replicates in control and 12 hrs post-IR group.


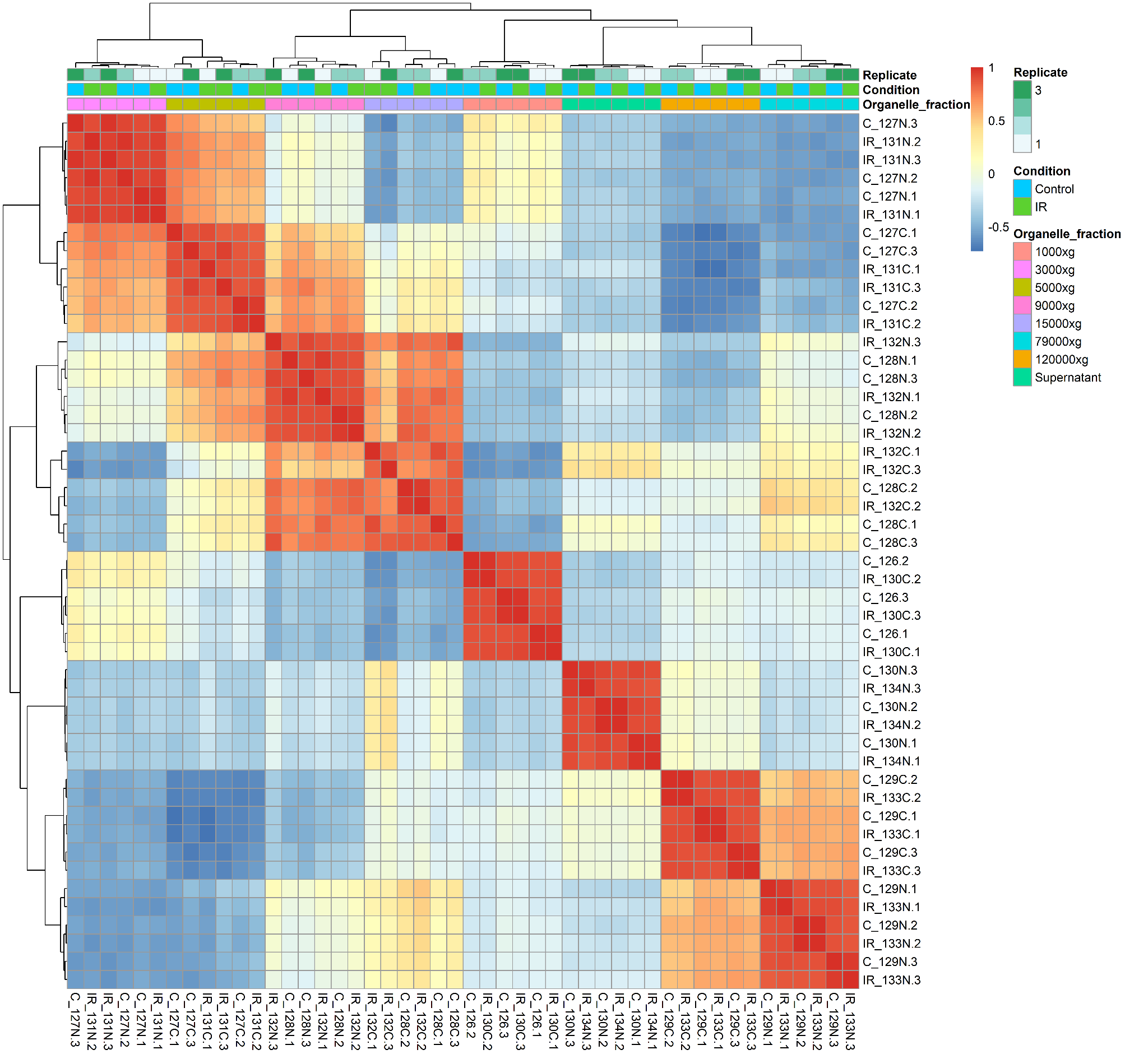


**Figure S6.** Pair-wise Pearson’s correlation matrix for each organellar lysate fraction for each LOPIT-DC experiment.


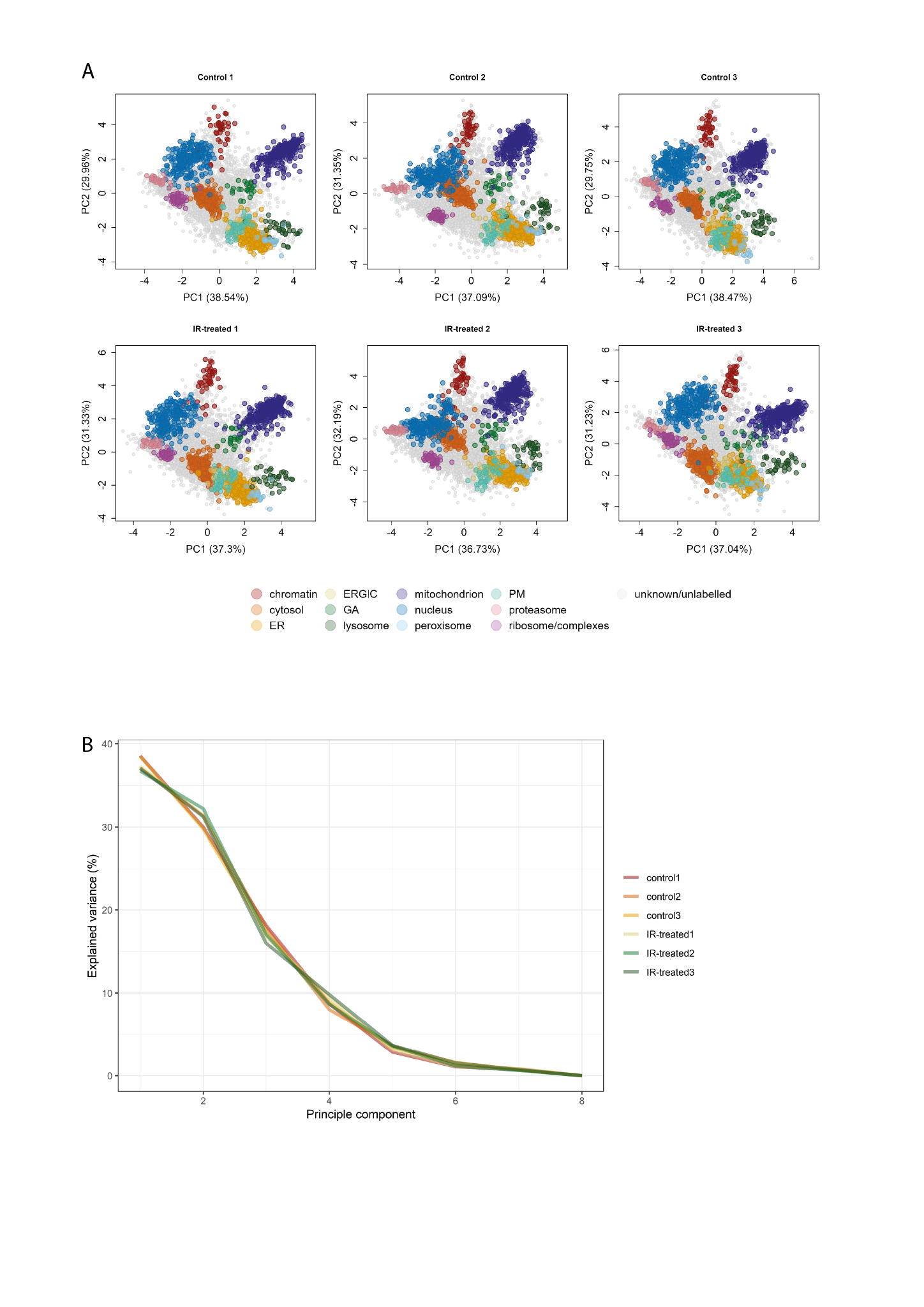


**Figure S7.** (A) Principal component analysis (PCA) plots for individual LOPIT-DC replicates. Each colour denotes the protein markers of 12 distinct subcellular compartments. (B) Corresponding skree plot showing percentage of explained variance across each principle component with each colour representing an individual LOPIT-DC replicate. (ER, endoplasmic reticulum; ERGIC, endoplasmic-reticulum–Golgi intermediate compartment; GA, Golgi apparatus; PM, plasma membrane).

**Figure S8.** Posterior predictive distributions (membership probability) across the 12 subceullar compartments for proteins identified as differentially localised and part of the exocyst complex in control vs IR-treated LOPIT-DC samples. (ER, endoplasmic reticulum; ERGIC, endoplasmic-reticulum–Golgi intermediate compartment; GA, Golgi apparatus; PM, plasma membrane).
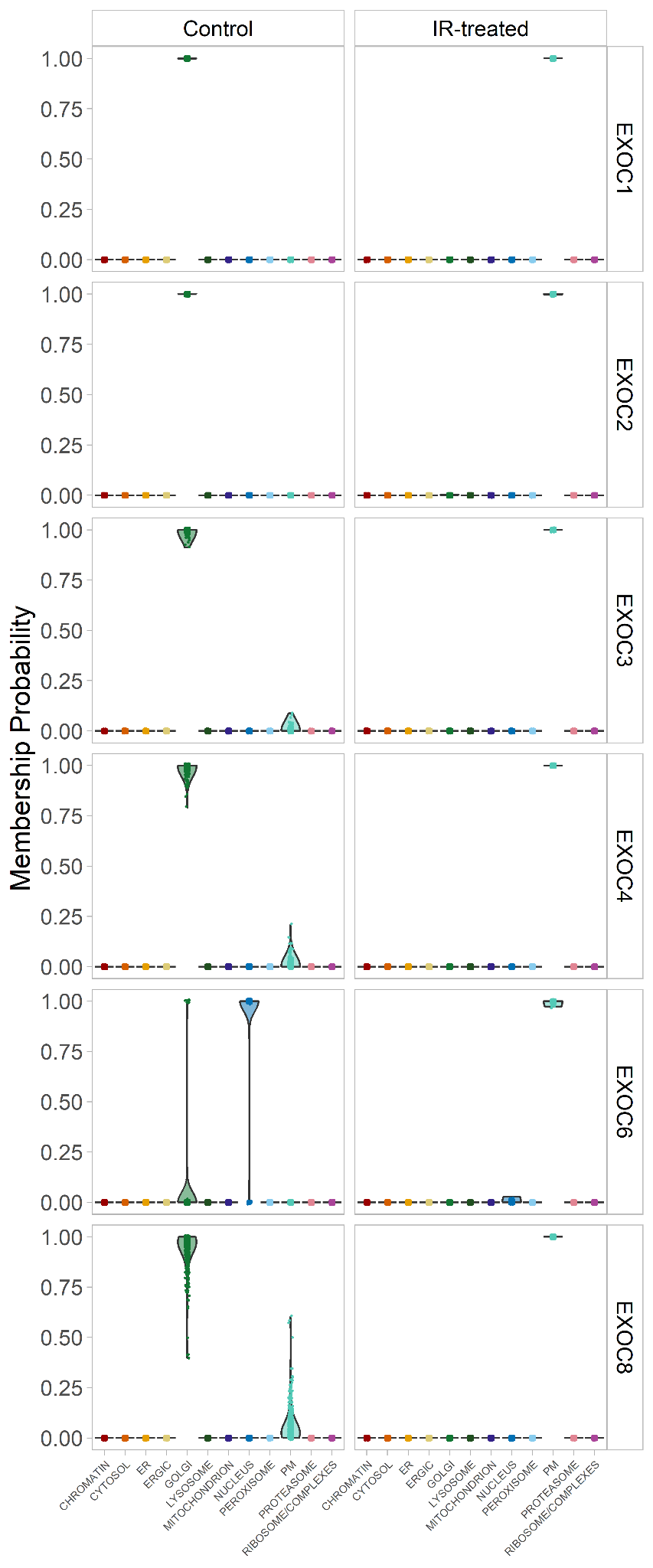


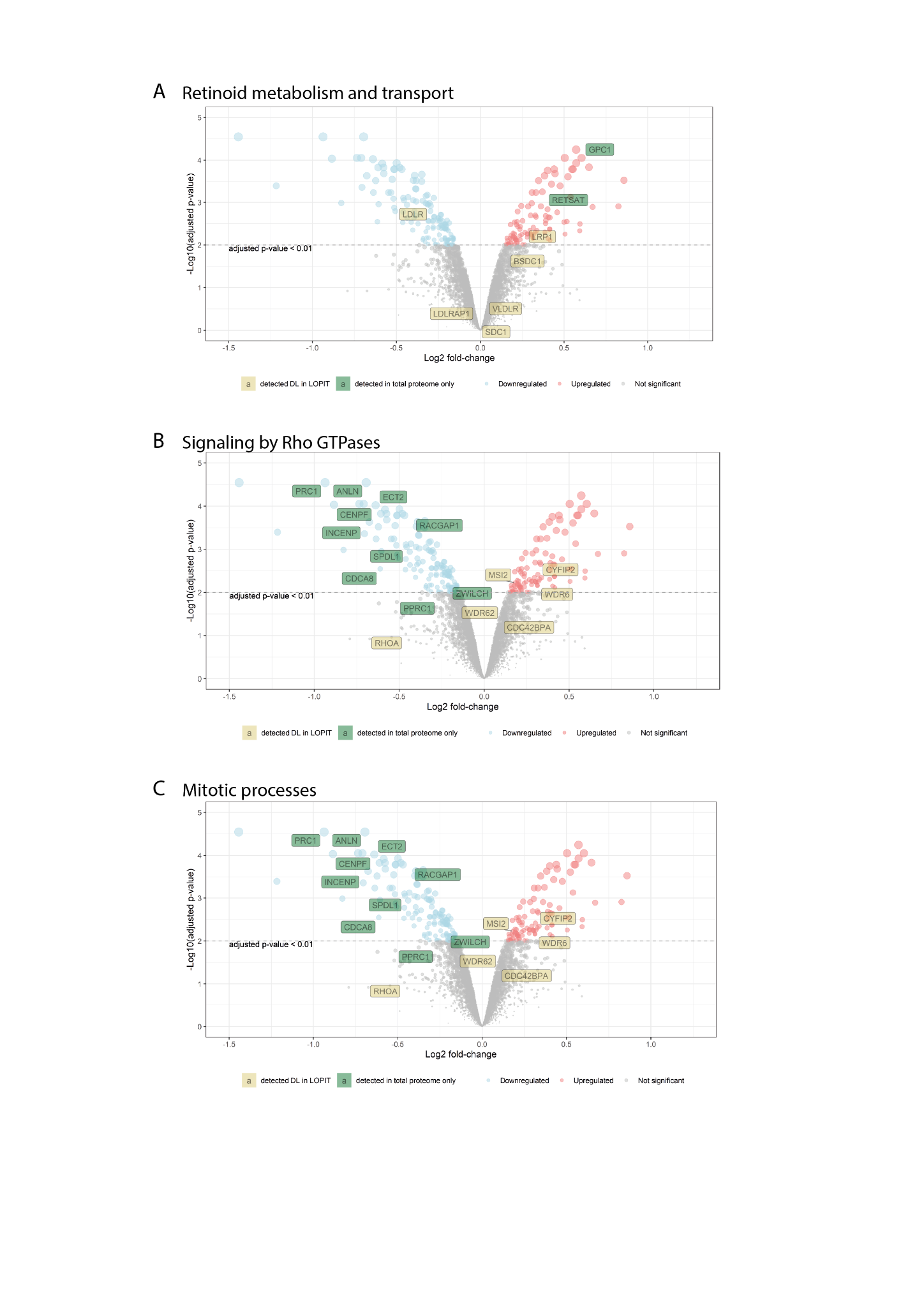


**Figure S9.** Volcano plot of differential expression analysis for the total proteomics data in control verus 12 hrs post-IR in A549 cells annotated with proteins involved in (A) retinoid metabolism and transport, (B) signalling of Rho GTPases, and (C) mitotic processes. Coloured points denote all proteins with FDR < 0.01 and coloured labels denote proteins detected as differentially localised (DL) in LOPIT-DC (eFDR < 0.05, yellow) or only detected in total proteomics (green).

**Figure S10.** Number of identified subcellular locations of “Enhanced” and “Supported” status proteins from the Human Protein Atlas (HPA) (Thul et al., 2017) against corresponding proteins within our study identified to have differentially expressed (gray) or localized (black) after IR exposure, according to an FDR < 0.05 or eFDR < 0.05, respectively.
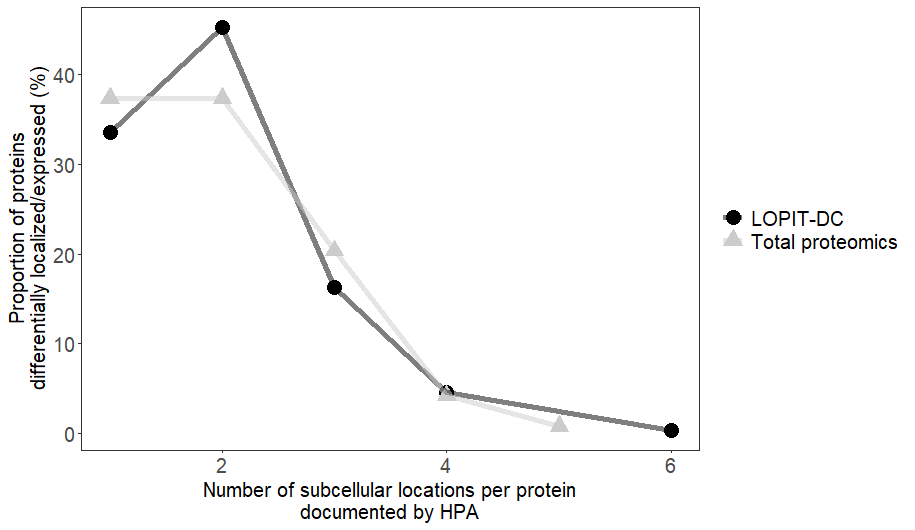


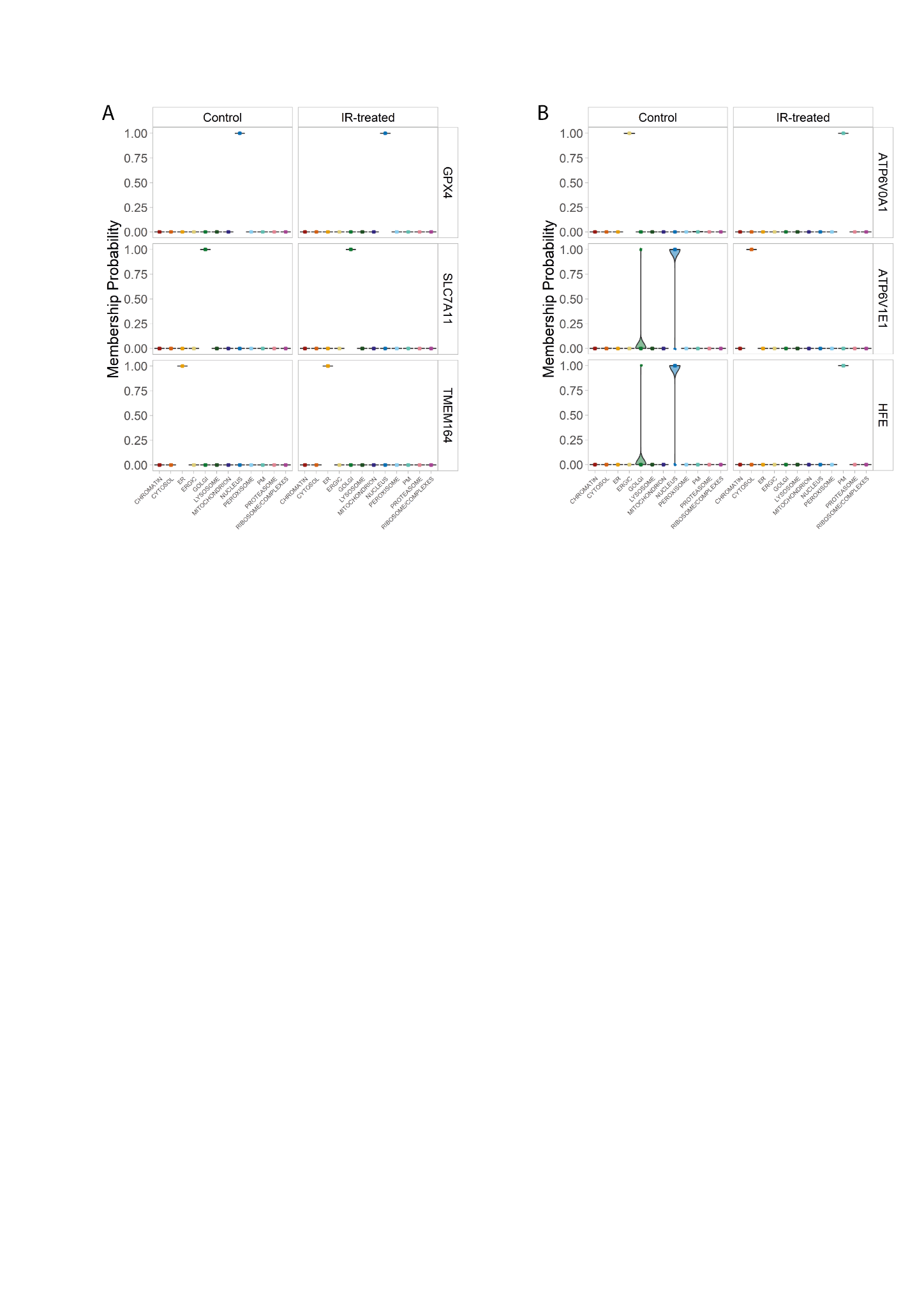


**Figure S11.** Posterior predictive distributions (membership probability) across the 12 subceullar compartments for proteins identified as (A) not differentially localised and part of ferroptosis pathway, and (B) differentially localised and part of iron uptake and transport in control vs IR-treated LOPIT-DC samples. (ER, endoplasmic reticulum; ERGIC, endoplasmic-reticulum–Golgi intermediate compartment; GA, Golgi apparatus; PM, plasma membrane).

**Figure S12.** Quantitative microscopy analysis to assess co-localisation of ferritin and LAMP1 immunostaining, and Membrite® staining.
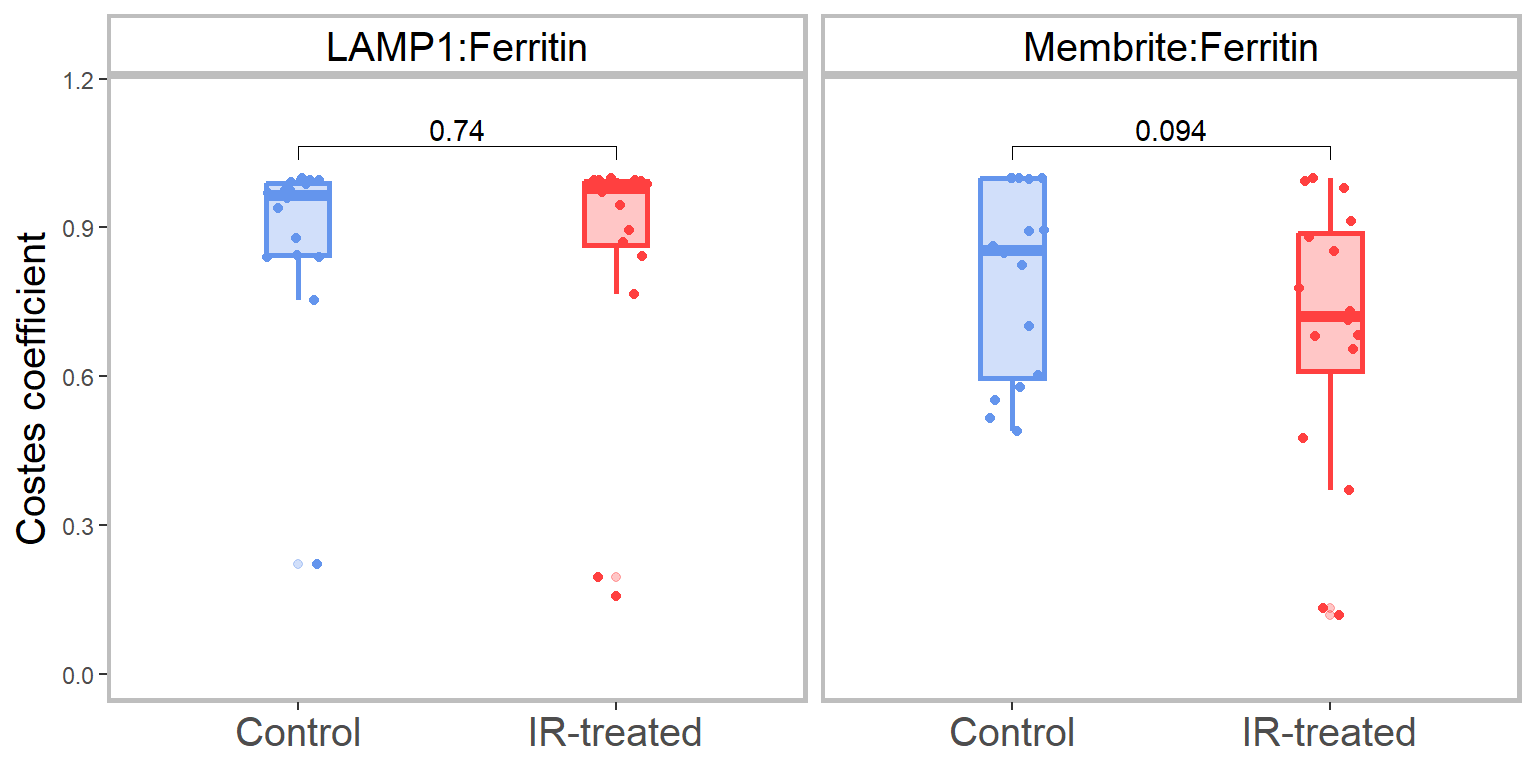


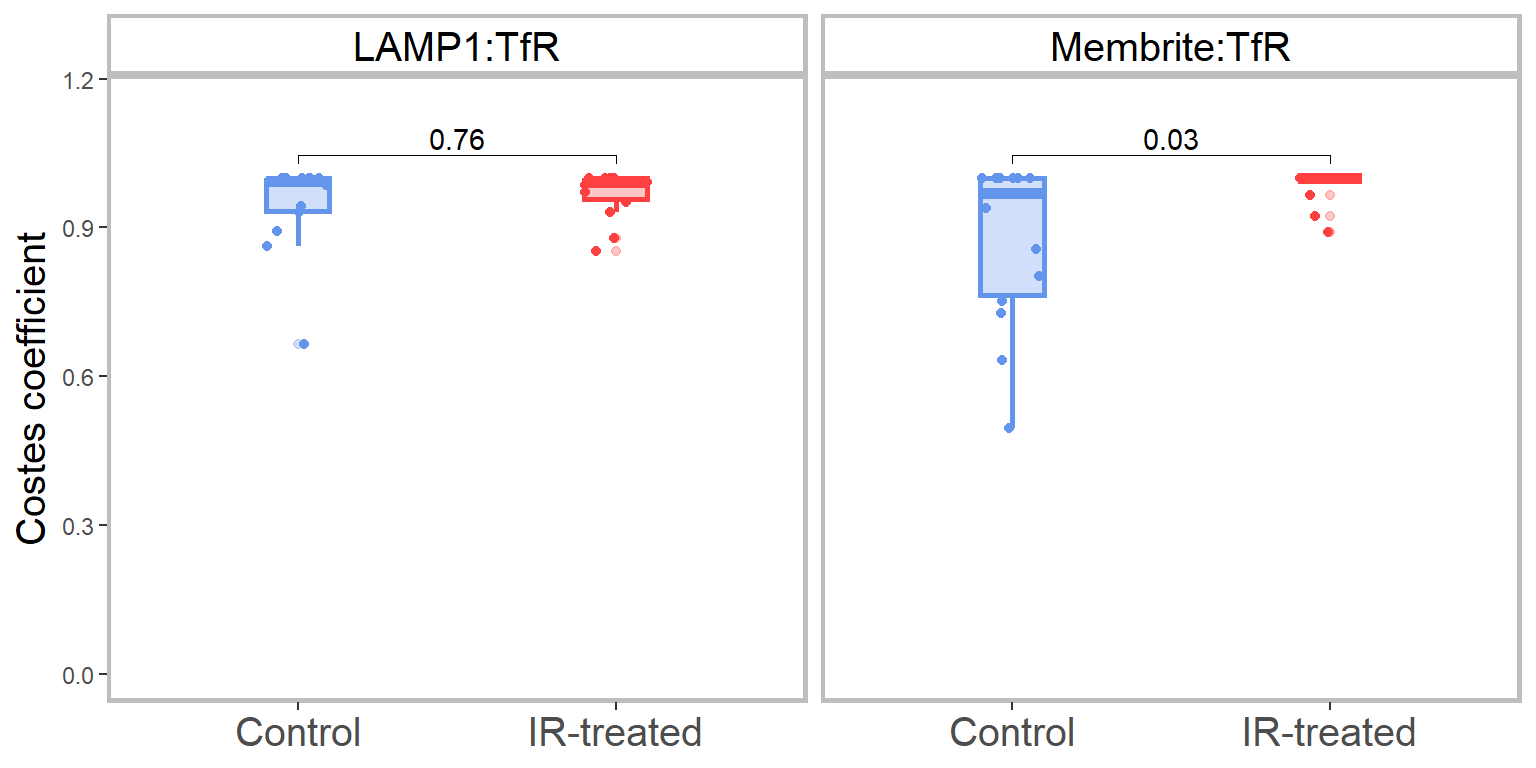


**Figure S13.** Quantitative microscopy analysis to assess co-localisation of LAMP1 and TFRC (TfR).
