# Supplementary material for: Global Proteomics Indicates Subcellular-Specific Anti-Ferroptotic Responses to Ionizing Radiation
Source: Mol Cell Proteomics. 2024 Nov 29;24(1):100888. doi: 10.1016/j.mcpro.2024.100888 (PMC11780130; doi:10.1016/j.mcpro.2024.100888)
Supplement: Supplemental Tables S1–S4 [file mmc4.docx]

**Table S1.** Spin speeds and duration of each centrifugation within the differential centrifugation steps of the LOPIT-DC workflow.

| **Fraction** | **Speed (xg)** | **Time (min)** |
| --- | --- | --- |
| Unlysed cells | 200 | 5 |
| Pellet 1 | 1,000 | 10 |
| Pellet 2 | 3,000 | 10 |
| Pellet 3 | 5,000 | 10 |
| Pellet 4 | 9,000 | 15 |
| Pellet 5 | 15,000 | 15 |
| Pellet 6 | 79,000 | 43 |
| Pellet 7 | 120,000 | 45 |
| Supernatant | - | - |

**Table S2.** Primary antibodies used in immunofluorescence microscopy experiment.

| **Target** | **Gene name** | **Product number** | **Manufacturer** | **Species** | **Used dilution/ concentration** |
| --- | --- | --- | --- | --- | --- |
| Ferroptosis suppressor protein 1 | AIFM2 | ab219986 | Abcam | rabbit | 0.25-2 μg/mL |
| Transferring receptor 1 | TFRC | ab84036 | Abcam | rabbit | 5 μg/mL |
| Ferritin | FTH1/FTL | ab75973 | Abcam | rabbit | 1:100 |
| Gamma-histone H2AX | ƴH2AX | ab22551 | Abcam | mouse | 2-4 μg/mL |
| Lysosome-associated membrane glycoprotein 1 | LAMP1 | ab25630 | Abcam | mouse | 5-10 μg/mL |

**Table S3.** P-values (*p*), effect sizes (*r*) and 95% confidence intervals (CI) for effect size of γH2AX foci measured via microscopy for control versus 12 hr post-IR comparison. Pairwise comparisons using t-test with Bonferroni adjustment were used (one-sided). Effect size (Pearson’s *r*) was calculated using z-statistic from the Wilcoxon test and bootstrapping used to calculate the 95% confidence interval (CI).

| **γH2AX foci location** | ***p* (adjusted)** | ***d* [95% CI]** |
| --- | --- | --- |
| Nuclei | 5.40e-13 | 0.58 [0.46-0.68] |
| Cytoplasm | 1.00 | 0.41 [0.23-0.58] |

**Table S4.** P-values (*p*), effect sizes (*d*) and 95% confidence intervals (CI) for effect size of cell cycle and cell death data collected via flow cytometry for control versus 12 hr post-IR comparison. Pairwise t-test with Bonferroni adjustment (two-sided). Effect size (Cohen’s *d*) was calculated with corresponding 95% confidence interval (CI) assuming equal variance in both groups.

| **Cell status/cell cycle phase** | ***p* (adjusted)** | ***d* [95% CI]** |
| --- | --- | --- |
| Viable | 1.00 | 0.13 [-1.48-1.73] |
| Apoptotic | 1.00 | 0.13 [-1.48-1.73] |
| Necrotic | 1.00 | -0.17 [-1.76-1.45] |
| G1 | 0.42 | -1.42 [-3.23-0.50] |
| G2 | 1.00 | -0.95 [-2.62-0.82] |
| S | 0.17 | 1.68 [-0.34-3.57] |
